# Supplementary material for: Phospholipid synthesis inside phospholipid membrane vesicles
Source: Commun Biol. 2022 Sep 27;5:1016. doi: 10.1038/s42003-022-03999-1 (PMC9515091; doi:10.1038/s42003-022-03999-1)
Supplement: Supplementary file 2 — Supplementary Information [file 42003_2022_3999_MOESM2_ESM.pdf]

Supplementary Information for
Phospholipid synthesis inside phospholipid membrane vesicles

Sumie Eto, Rumie Matsumura, Yasuhiro Shimane, Mai Fujimi, Samuel Berhanu, Takeshi Kasama, Yutetsu Kuruma

Corresponding author: Yutetsu Kuruma
Email: ykuruma@jamstec.go.jp

This PDF file includes:

Supplementary Figures 1 to 20
Supplementary Table 1 to 10
Supplementary Text 1
Supplementary References

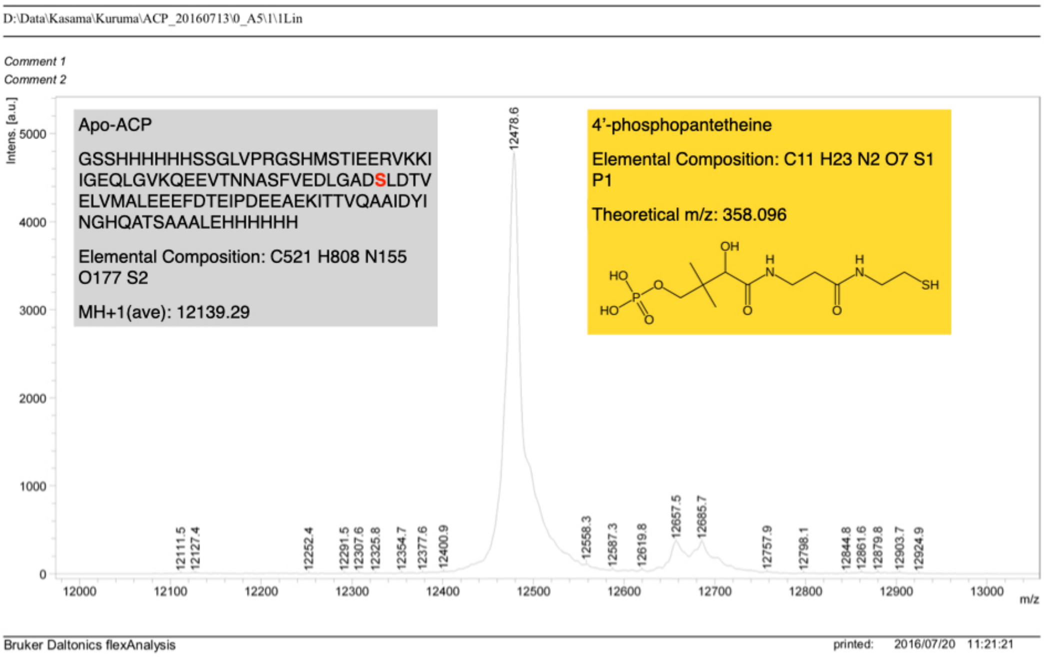

Supplementary Figure 1. MALDI-TOF-MS data confirming holo-ACP in the purified material. Acyl carrier protein (ACP) was overexpressed in *E. coli* BL21 (DE3) strain together with 4'-phosphopantetheinyl transferase (SFP). The expressed apo-type ACP (apo-ACP) was post-translationally modified by SFP to generate holo-type ACP (holo-ACP). The holo-ACP was purified by Ni-NTA column, then by monoQ column. The purified holo-ACP was analyzed by MALDI-TOF-MS. Amino acid sequence of apo-ACP missing the first methionine is described in the gray color box and the serine residue modified with 4’-phosphopantetheine is shown by red color. The structure and theoretical *m/z* value of 4’-phosphopantetheine are shown in the yellow color box. The *m/z* value of holo-type ACP is given as *12478.6*.

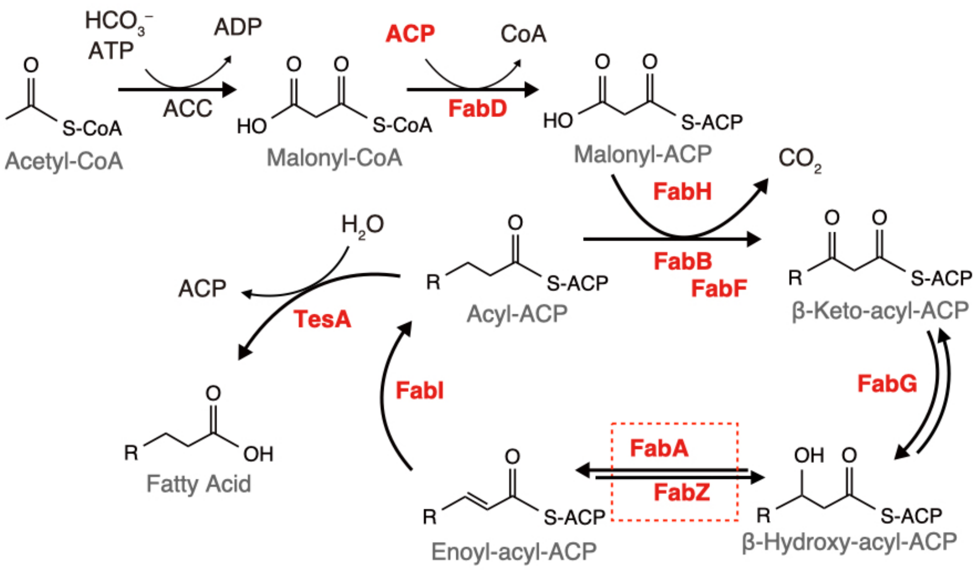

Supplementary Figure 2. Fatty acid synthesis pathway. Enzymes purified for the *in vitro* fatty acid synthesis system are shown in red. The dehydration step by FabA or FabZ is shown by a red box with the dashed line. All enzymes are originated from *E. coli* and purified individually. CoA: coenzyme A, ACP: acyl carrier protein, TesA: thioesterase 1, ATP: adenosine triphosphate.

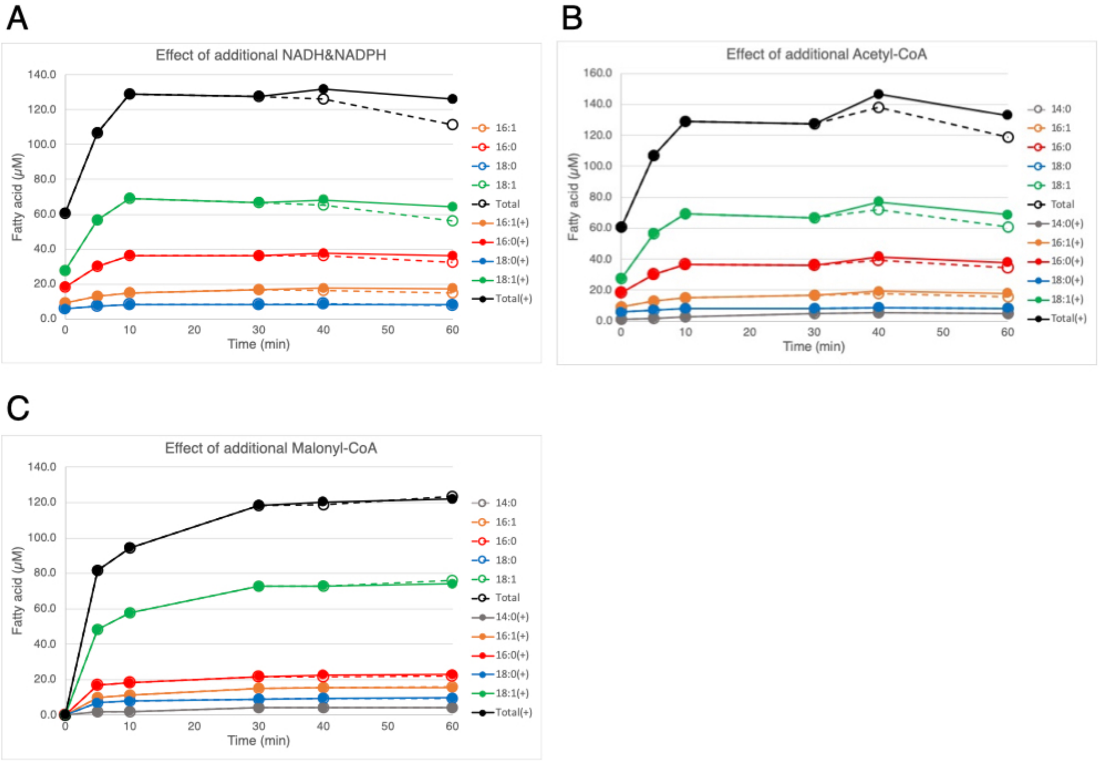

Supplementary Figure 3. Time-course analysis of fatty acid synthesis by the additional supply of factors. In vitro fatty acid synthesis was performed by mixing FabA (1 μM), FabB (1 μM), FabD (10 μM), FabF (10 μM), FabG (1 μM), FabH (1 μM), FabI (10 μM), FabZ (1 μM), ACP (30 μM), TesA (30 μM), NAD(P)H (5 mM), ^{13}C-acetyl-CoA (0.5 mM), and ^{13}C-malonyl-CoA (1.25 mM). The reaction mixtures were incubated at 37 ˚C and collected at each time point for the quantification by LC/MS. During the reactions, NAD(P)H (A), ^{13}C-acetyl-CoA (B), or ^{13}C-malonyl-CoA (C) were additionally supplied at the 30 minutes as the same amount as the initial concentration (the traces of filled circles and solid lines). For the comparison, the buffer of each material was supplied as the same volume (the traces of empty circles and dashed lines). Types of fatty acids and total yield are shown on the right side of each graph.

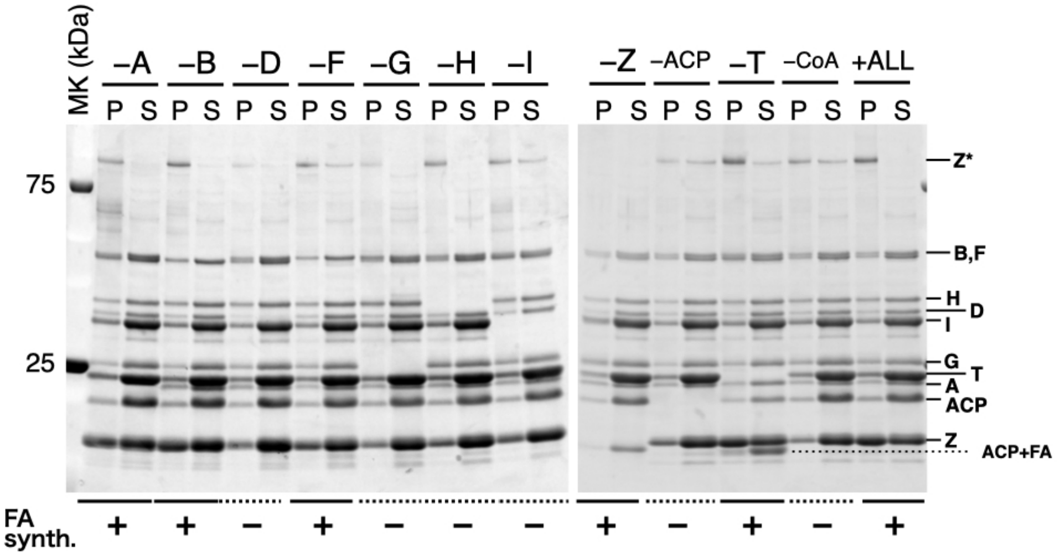

Supplementary Figure 4. Solubility tests of the fatty acid synthesis enzymes after reacting the reaction solutions lacking each component. Fatty acid syntheses were performed in the absence of each component at 30 ˚C for 30 min. The resulting reaction solutions were centrifuged to separate the component proteins into soluble (S: supernatant) or non-soluble (P: precipitate) fractions, then analyzed by SDS-PAGE. The images were obtained by staining the gel with a fluorescent reagent, Oriole (Bio-Rad). The omitted component is indicated at the top of the gel. When an essential or non-essential component was omitted, fatty acid synthesis (FA synth.) − or + is indicated at the bottom of the gel, respectively. The positions of each enzyme and molecular markers (MK) are indicated at the right and left sides of the gel, respectively. −CoA indicates acetyl-CoA and malonyl-CoA were omitted. +All indicates all enzymes and substrates were presented. Z* shows a multimer of FabZ.

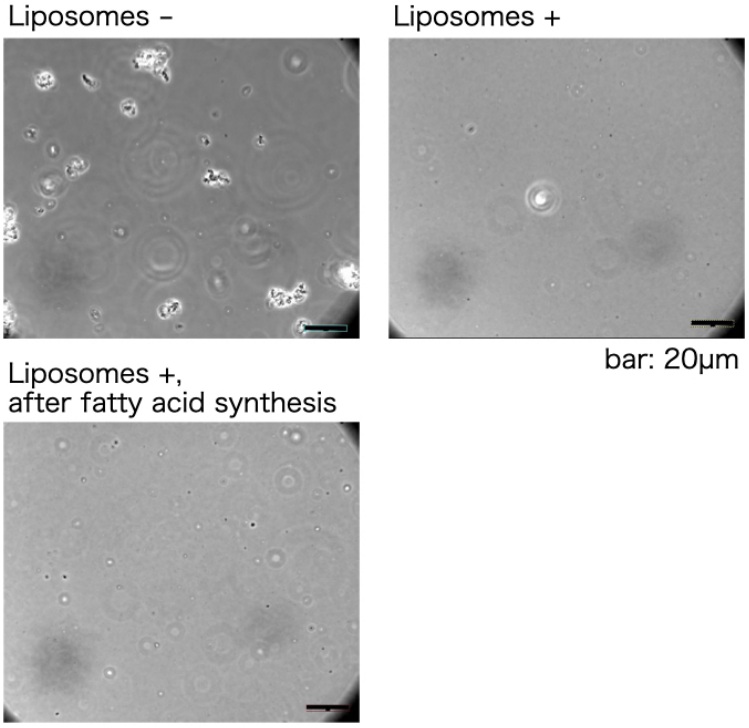

Supplementary Figure 5. The effect of liposomes on the formation of aggregates in the reaction solution of fatty acid synthesis. *In vitro* fatty acid syntheses were performed at 37 ˚C for 30 min in the presence or absence of liposomes. The resulting reaction solutions were observed by optical microscopy (Olympus IX73) equipped with an x100 phase-contrast objective lens. Images were obtained by CMOS camera (Zyla, Andor) and MetaMorph software. The reaction solution which was supplied liposomes after the fatty acid synthesis is also shown. Scale bar: 20 μm.

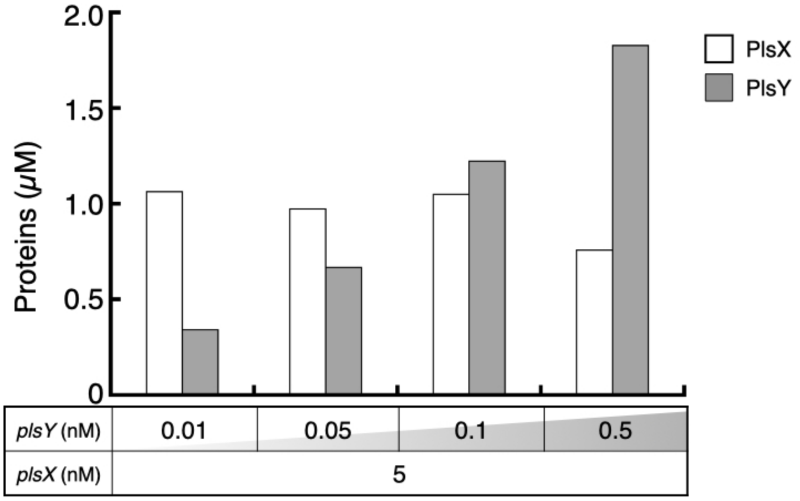

Supplementary Figure 6. Yields of co-synthesized PlsX and PlsY in the PURE system. Cell-free protein syntheses were performed at 37 ˚C for three hours. The reactions were initiated by the addition of DNAs. Concentrations of the added *plsX* and *plsY* genes are shown at the bottom of the graph. Note that both genes contain additional sequences for six histidines at the C-terminus of the proteins. After the reaction, the reaction mixtures were processed by SDS-PAGE and western blotting using an anti-6His antibody. The concentrations of synthesized PlsX and PlsY were quantified by comparing the band intensities of the defined purified proteins. The molecular sizes of PlsX and PlsY are 38 and 22 KDa, respectively.

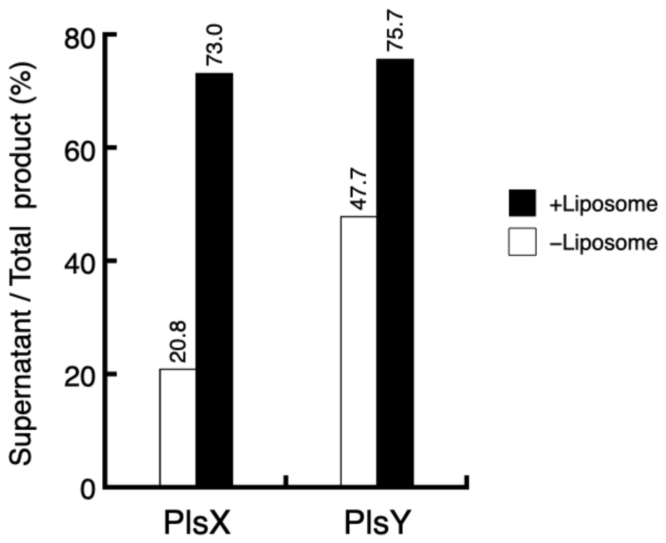

Supplementary Figure 7. Solubility of PlsX and PlsY synthesized in the PURE system in the presence or absence of liposomes. PlsX and PlsY containing a histidine-tag sequence at the C-terminus of each protein were synthesized as described in the legend of Supplementary Figure 6, in the presence or absence of liposomes consisting of 50 % POPC and 50 % POPG. After the syntheses, the reaction mixtures were mixed with the same amount of 2.4 M sucrose solution, then 0 M sucrose solution was supplied over the sucrose layer in a centrifugation tube. The resulting solutions were ultracentrifuged at 385,000 × g for 3 hours at 4 ˚C. The supernatants were collected and transferred into fresh tubes, and the precipitants were dissolved by 0 M sucrose solution. Proteins in both supernatant and precipitant fractions were concentrated by trichloroacetic acid precipitation and analyzed by SDS-AGE and western-blotting using an anti-histidine tag antibody. The solubility was derived by dividing the band intensity of the obtained supernatant fraction by the band intensity of the sum of supernatant and precipitant fractions.

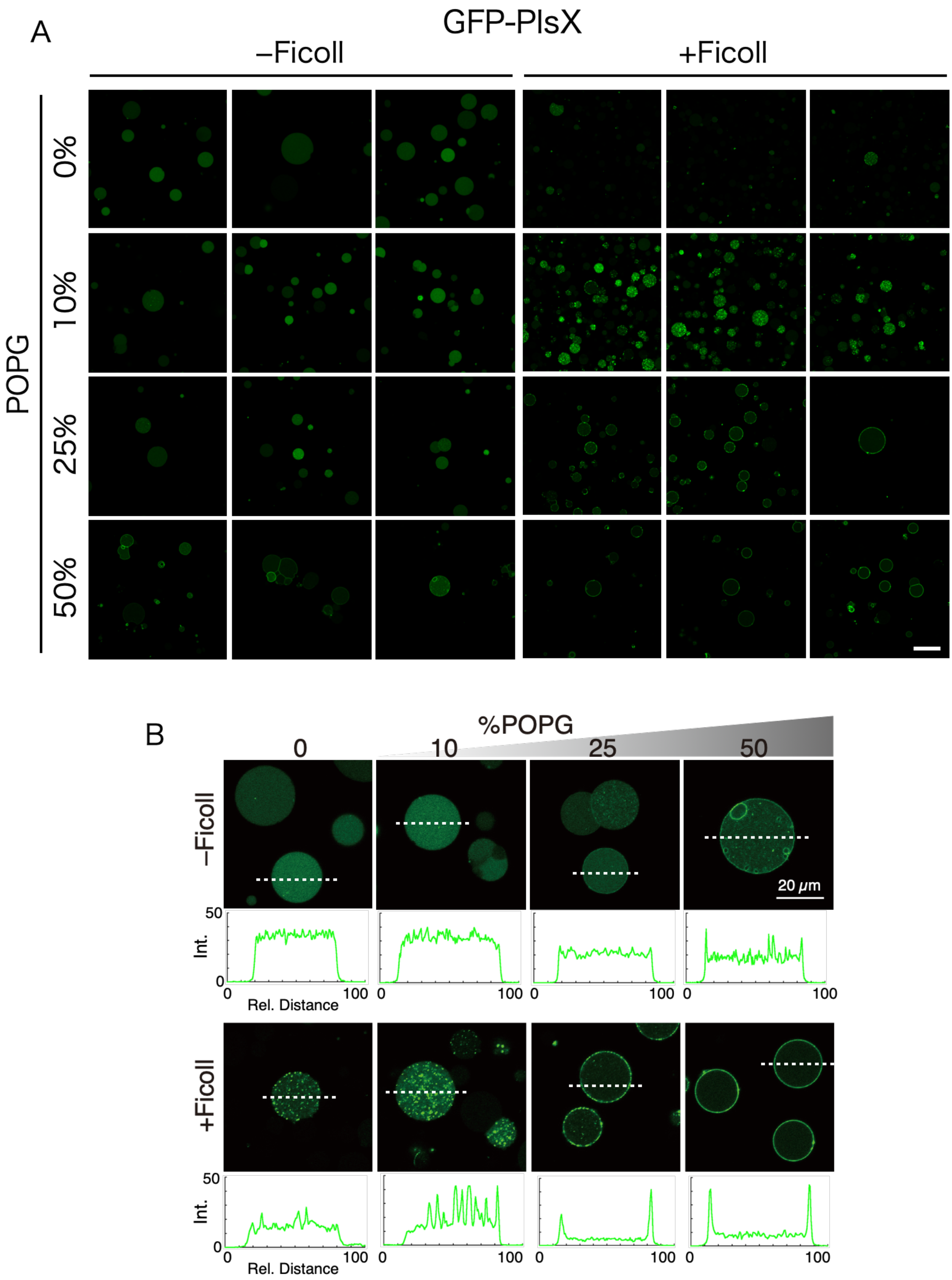

Supplementary Figure 8. Membrane localization of GFP-PlsX synthesized in giant vesicles in the presence or absence of Ficoll. (A) PlsX fused with GFP on the N-terminus was synthesized by the PURE system inside giant vesicles (GVs). The ratios of anacidic

phospholipid, POPG, in the lipid composition of the GV are described on the left side of the images. The protein syntheses were performed in the presence (+Ficoll) or absence (–Ficoll) of Ficoll PM70, which was used for mimicking the molecular crowd in the cytoplasm. Images were obtained by Nikon confocal microscopy system (A1R). Scale bar: 40 μm. **(B)** Plot profiles of GFP-PlsX. The intensities of fluorescent signals on the broken lines were measured by ImageJ software and shown as traces at the bottom of each image. The X and Y-axis represent relative distance and fluorescent intensity, respectively.

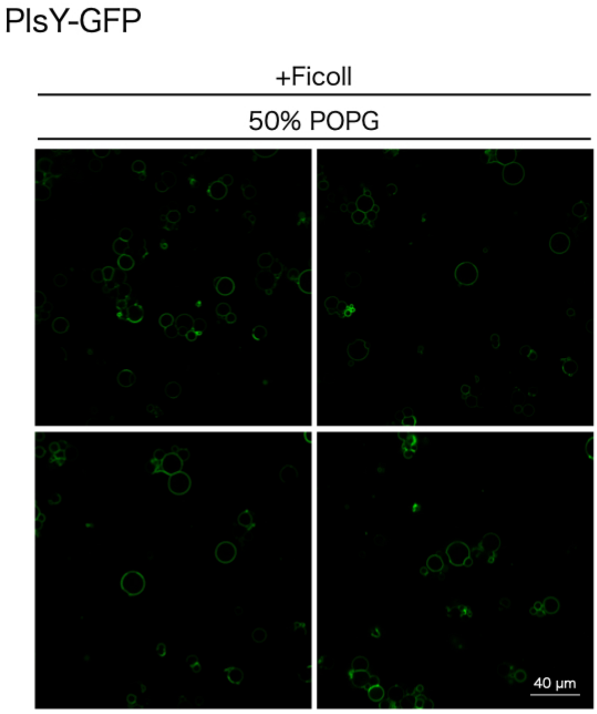

Supplementary Figure 9. Membrane localization of PlsY-GFP synthesized inside giant vesicles in the presence of Ficoll. PlsY fused with GFP at the C-terminus was synthesized inside giant vesicles and observed by confocal microscopy as described in Supplementary Figure 8 legend. Proteins were synthesized in the presence of Ficoll. Lipid composition of giant vesicles was POPC 50 mol%: POPG mol%. Scale bar: 40 µm.

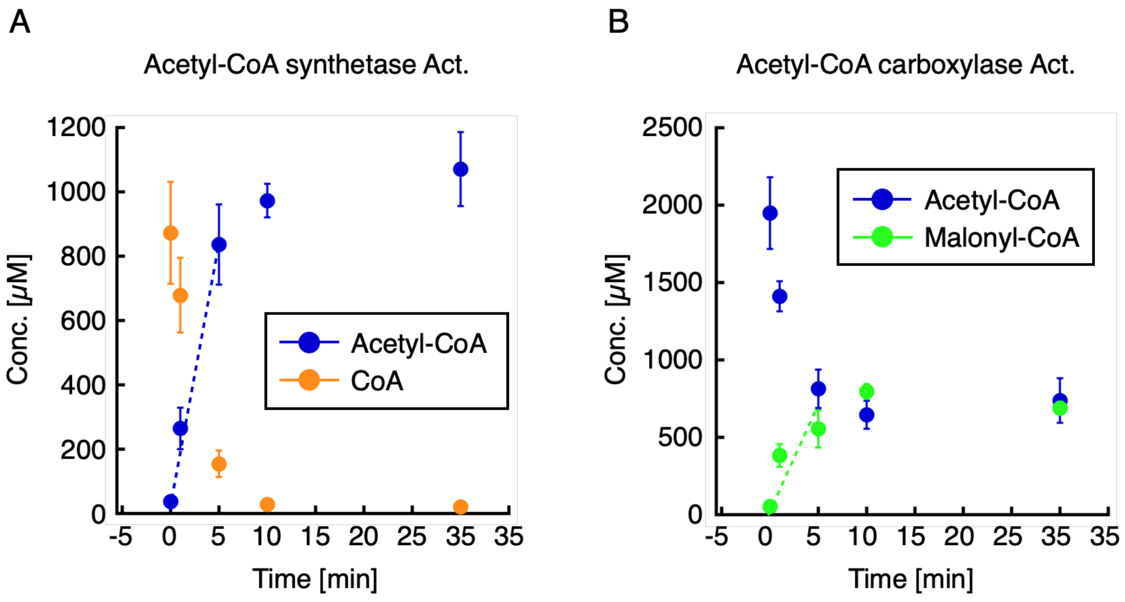

425 umol/min/mg 300 umol/min/mg

Supplementary Figure 10. Kinetic analysis of acetyl-CoA (ACS) synthetase and acetyl-CoA carboxylase (AccABCD). (A) The enzymatic activity of ACS was measured by quantifying the amount of synthesized acetyl-CoA in the reaction mixture. The reaction mixture was prepared as 0.4 mg/ml ACS, 1 mM CoA, 1 mM ATP, 1 mM K(OAc)2, and PURE*frex* Sol. I. Reaction was carried out at 37 ˚C. The collected samples at each time point were diluted 100-folds with 70 % MeOH and centrifuged at 150,000 rpm for 5 min. The resulting supernatant was analyzed by Shimadzu LCMS-2020 equipped with L-column3 (CERI, JAPAN). The obtained area data of acetyl-CoA or CoA were converted into mg using a standard curve obtained by measuring the series of a defined amount of acetyl-CoA or CoA. The dashed line indicates the slope between 0-5 min of acetyl-CoA, and the calculated initial velocity was shown at the bottom of the graph. (B) The enzyme activity of AccDA in the presence of AccBC was measured as the same as in (A). The reaction mixture was prepared as 0.4 mg/ml AccDA, 0.76 mg/ml AccBC, 2 mM acetyl-CoA, 1 mM ATP, 10 mM $KHCO_3$, and PURE*frex* Sol. I. The dashed line indicates the slope between 0-5 min of malonyl-CoA, and the calculated initial velocity was shown at the bottom of the graph. Error bars indicate the standard deviation of at least triplicate measurements.

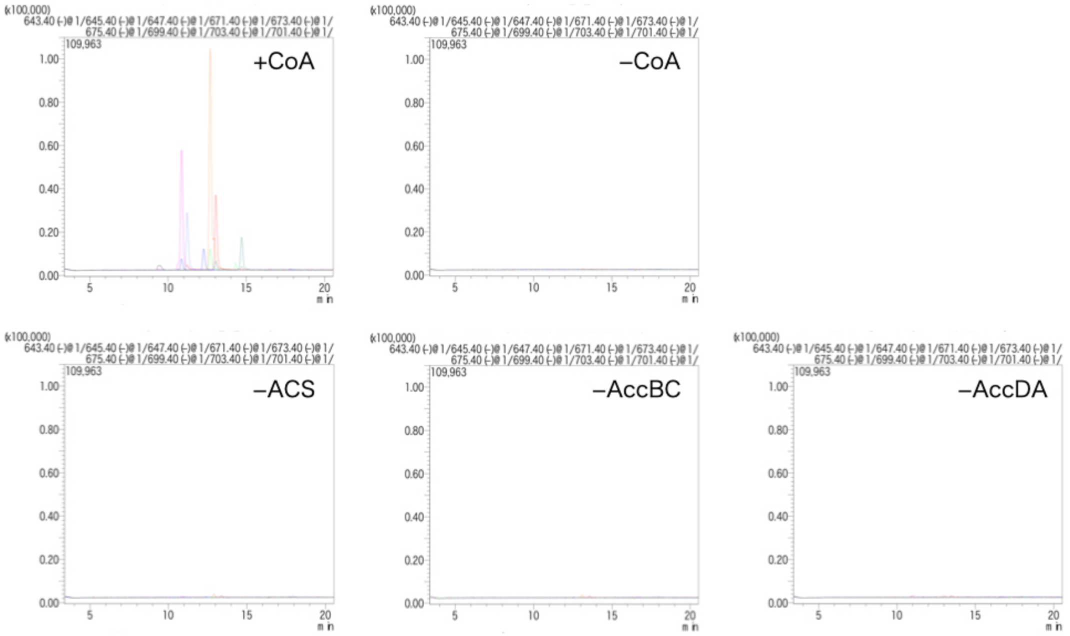

Supplementary Figure 11. Cell-free phosphatidic acid (PA) synthesis with recycling CoA. PA syntheses were performed in the presence (+CoA) or absence (−CoA) of the substrate in the cell-free system synthesizing three acyltransferases (PlsX, PlsY, and PlsC) as described in Supplementary Figure 6 legend. During the protein syntheses, fatty acid binding proteins, acetyl-CoA synthetase (ACS), acetyl-CoA carboxylase (AccBC and AccDA), and liposomes (POPC 50 mol% and POPG 50 mol%) were supplied. After the protein syntheses, PA syntheses were demonstrated by adding CoA and NAD(P)H. After the PA synthesis, phospholipid products were extracted with methanol and analyzed by a LC/MS. To examine the dependency of each factor, the reaction missing CoA (−CoA), ACS (−ACS), AccBC (−AccBC), or AccDA (−AccDA) were also performed. The peak data of mass spectrometry monitoring the PA products, which are unlabeled with stable isotope carbon, in negative-ion mode are shown.

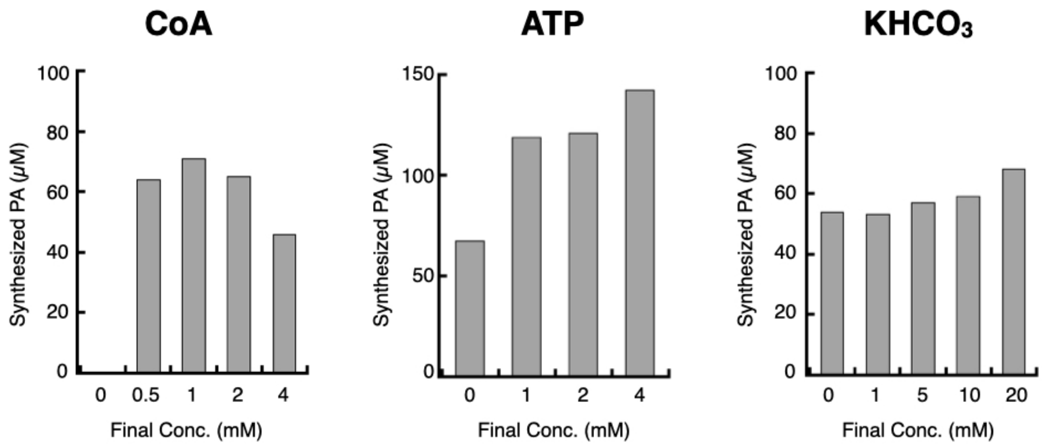

Supplementary Figure 12. Titration of CoA, ATP, or $KHCO_3$ in the PA synthesis reaction recycling CoA. After cell-free syntheses of PlsX, PlsY, and PlsC, various amounts of CoA, $KHCO_3$, and ATP were mixed with the cell-free mixture then the PAs syntheses were performed at 37 ˚C for 30 min by adding 8 mM NAD(P)H. The PA products were extracted with methanol and quantified by LC/MS.

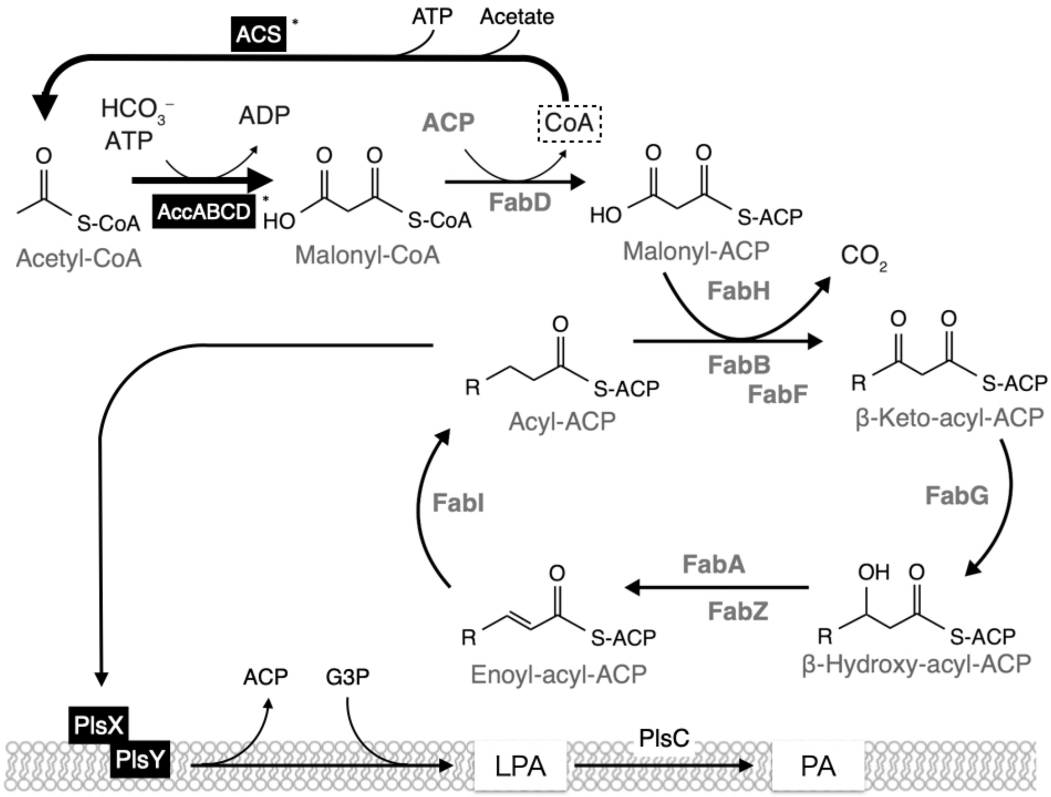

Supplementary Figure 13. Schematic map of the cell-free phospholipid synthesis system including a CoA recycling system. Acyl-CoA synthetase (ACS) and acetyl-CoA carboxylase ABCD (AccABCD) were added into the cell-free phospholipid synthesis system to recycle the reacted CoA and to generate acetyl-CoA and malonyl-CoA. acetate and HCO_3^- were consumed as substrates of ACS and AccABCD, respectively. Both reactions require ATP as an energy.

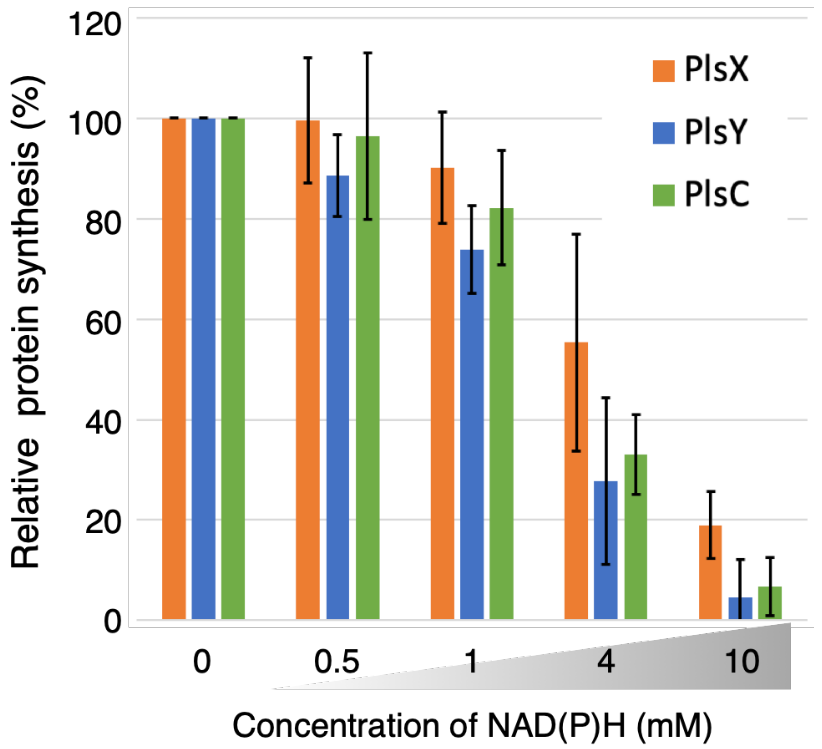

Supplementary Figure 14. Effect of NAD(P)H on protein synthesis in the cell-free system. PlsX, PlsY and PlsC were synthesized in the PURE system containing the various concentration of NAD(P)H (mixture of NADH and NADPH). All proteins were containing 6 histidine-tag (HisTag) at the C-terminus and synthesized in the presence of liposomes and DnaKJE chaperon. The synthesized proteins were analyzed by SDS-PAGE followed by western blotting using an anti-HisTag antibody. The bands of products were visualized by a chemiluminescence imaging reagent (ECL, Amersham) for quantification. The data were obtained from three independent experiments. Error bars indicate the standard deviation of triplicate measurements.

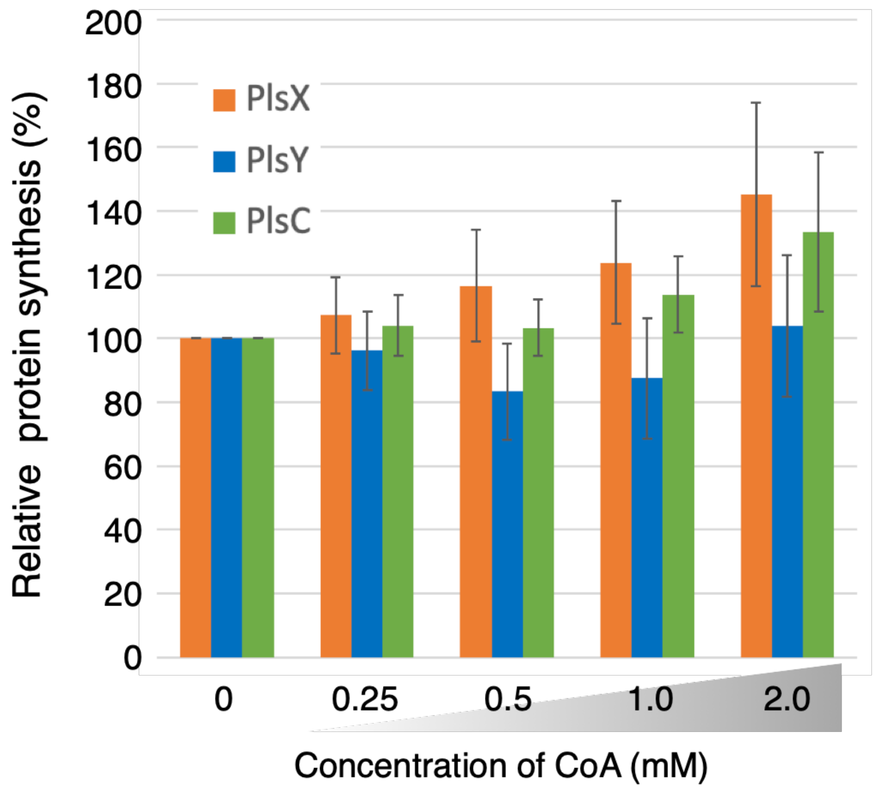

Supplementary Figure 15. Effect of CoA on protein synthesis in the cell-free system. PlsX, PlsY, and PlsC were synthesized in the PURE system containing the various concentration of CoA. All proteins were containing 6 histidine-tag (HisTag) at the C-terminus and synthesized in the presence of liposomes and DnaKJE chaperon. The synthesized proteins were analyzed by SDS-PAGE followed by western blotting using an anti-HisTag antibody. The bands of products were visualized by a chemiluminescence imaging reagent (ECL, Amersham) for quantification. The data were obtained from three independent experiments. Error bars indicate the standard deviation of triplicate measurements.

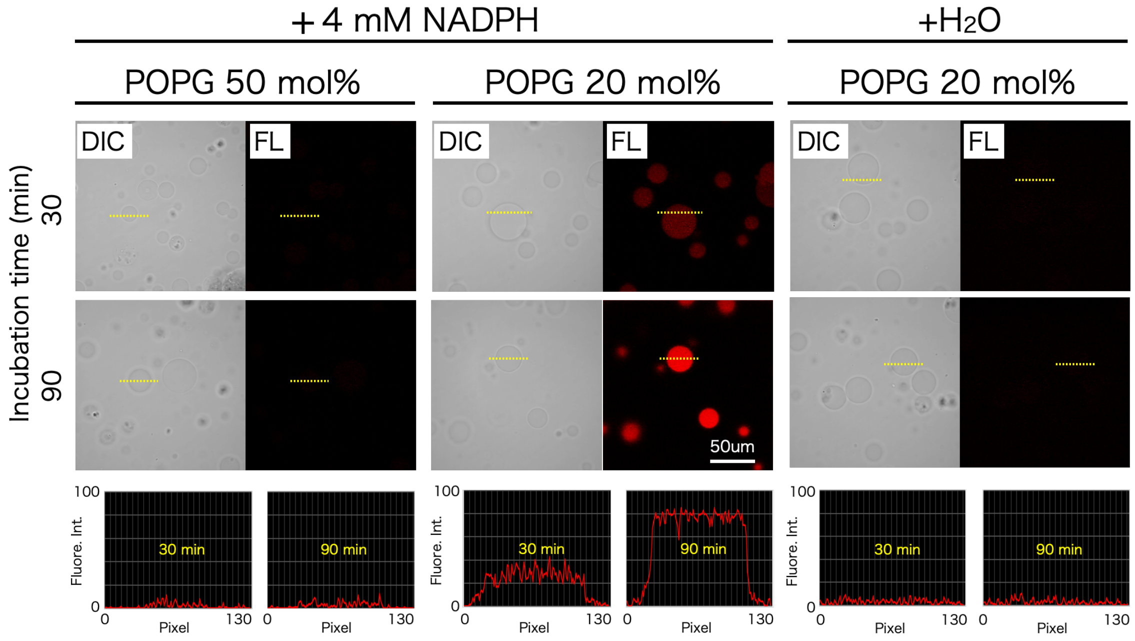

Supplementary Figure 16. Membrane permeability test of NADPH. The fluorometric probe regent and electron mediator of the NADP+/NADPH Assay Kit (Cell Biolabs, INC.) were diluted in 50 mM Hepes-KOH (pH 7.5) buffer containing 200 mM sucrose according to the manufacturer's instruction, and encapsulated in giant vesicles composed of 50 mol% POPC and 50 mol% POPG, or 80 mol% POPC and 20 mol% POPG. After the formation of vesicles, 4 mM NADPH or H_2O was introduced to the outside of the vesicles, followed by incubation at 37 ˚C for 30 or 90 minutes. The resulting vesicles were observed by confocal microscopy (Nikon A1R system) with a DIC unit or a 561 nm laser (FL). The fluorescent intensities of the vesicles were measured by a plot profiling measurement of ImageJ software. The exposure and contract of the DIC images were adjusted to easier to see. All FL images were obtained with the same laser power and gain (HV). DIC: differential interference contrast, FL: fluorescence.

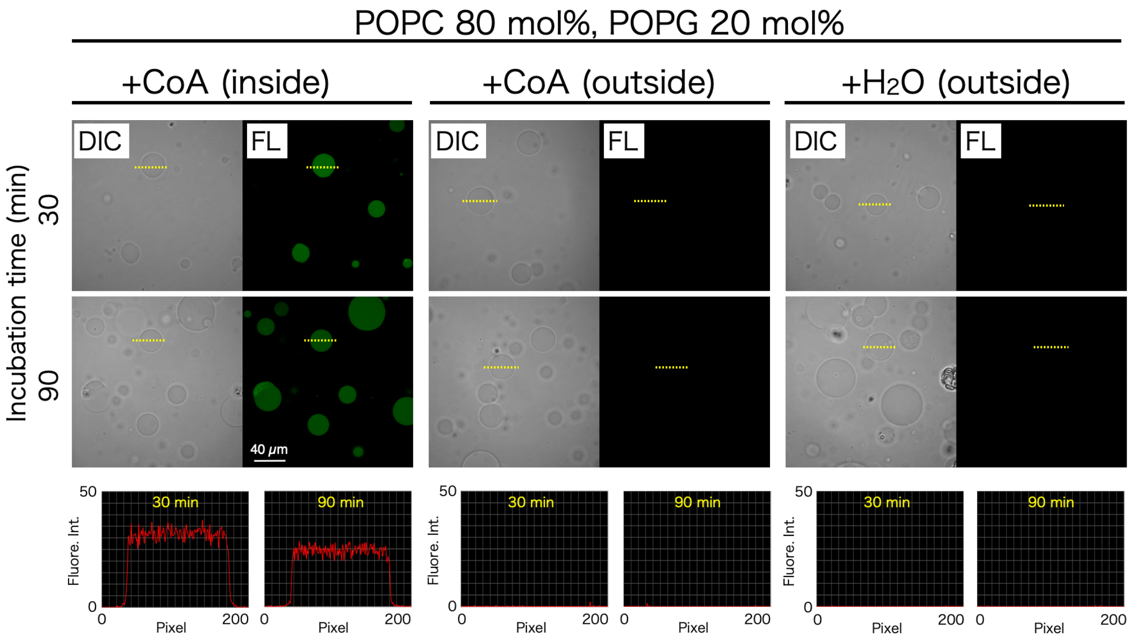

Supplementary Figure 17. Membrane permeability test of CoA. The fluorometric probe regent of the CoA Assay Kit (Sigma-Aldrich) was encapsulated in giant vesicles together with the buffer attached to the product and 200 mM sucrose. The lipid composition of the vesicles was 80 mol% POPC and 20 mol% POPG. After the formation of vesicles, 0.5 mM CoA was introduced into the outer solution (the same buffer as the inner solution missing the fluorescent probe and sucrose) containing 200 mM glucose, followed by incubation at 37 ˚C for 30 or90 minutes. As a positive control, 0.5 mM CoA was encapsulated inside the vesicles in advance. On the other hand, as a negative control, H_2O was added into the outer solution instead of CoA. The resulting vesicles were observed by confocal microscopy (Nikon A1R system) with a DIC unit or a 488 nm laser (FL). The fluorescent intensities of the vesicles were measured by a plot profiling measurement of ImageJ software. The exposure and contract of the DIC images were adjusted to easier to see. All FL images were obtained with the same laser power and gain (HV). DIC: differential interference contrast, FL: fluorescence.

Reaction condition
Vesicle: POPC 80 mol%, POPG 20 mol%
Lipid synth. time: 1 - 9 hours @ 37 ˚C
NADPH conc.: 8 mM
Glucose at the outer sol.: 300 mM
GFP-Spo conc.: 0.85 mg/mL (working conc.)

Nikon A1R Confocal Microscopy
FITC
HV(GaAsP):35
Offset: 0
Laser (488): 3.5
Scan size: 1024
Scan speed: 0.5 frame/sec (Pixcel Dwell: 0.97 u sec)

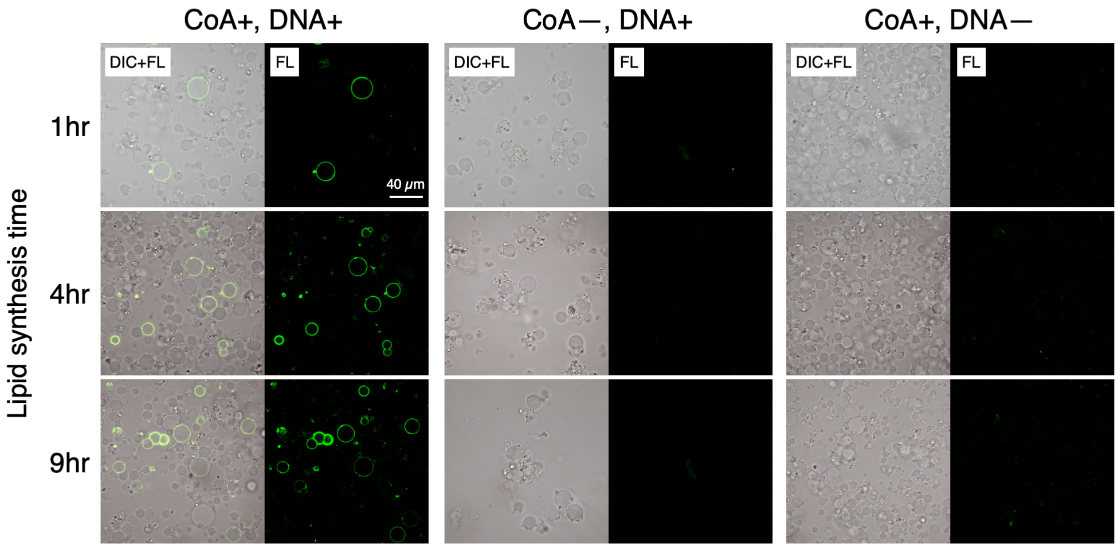

Supplementary Figure 18. Confocal microscopy image of the artificial cells synthesizing phospholipids. Artificial cells were prepared by encapsulating the cell-free mixture (PURE system) together with fatty acid synthesis enzymes, as shown in Supplementary Table 10, inside giant vesicles composed of 80 mol% POPC and 20 mol% POPG. After the three acyltransferases synthesis, the internal phospholipid synthesis reaction was initiated by the addition of 8 mM NADPH (working concentration), which is permeable to the vesicle membrane, and carried out for 1, 4, or 9 hours. The reaction without CoA or DNAs encoding the acyltransferases was also performed as a control. The resulting artificial cells were mixed with 0.85 mg/ml GFP-Spo (working concentration), a probe protein for phosphatidic acid, then observed by confocal microscopy (Nikon A1R system) with a 488 nm laser (FL) or DIC overlaying FL. DIC: differential interference contrast, FL: fluorescence.

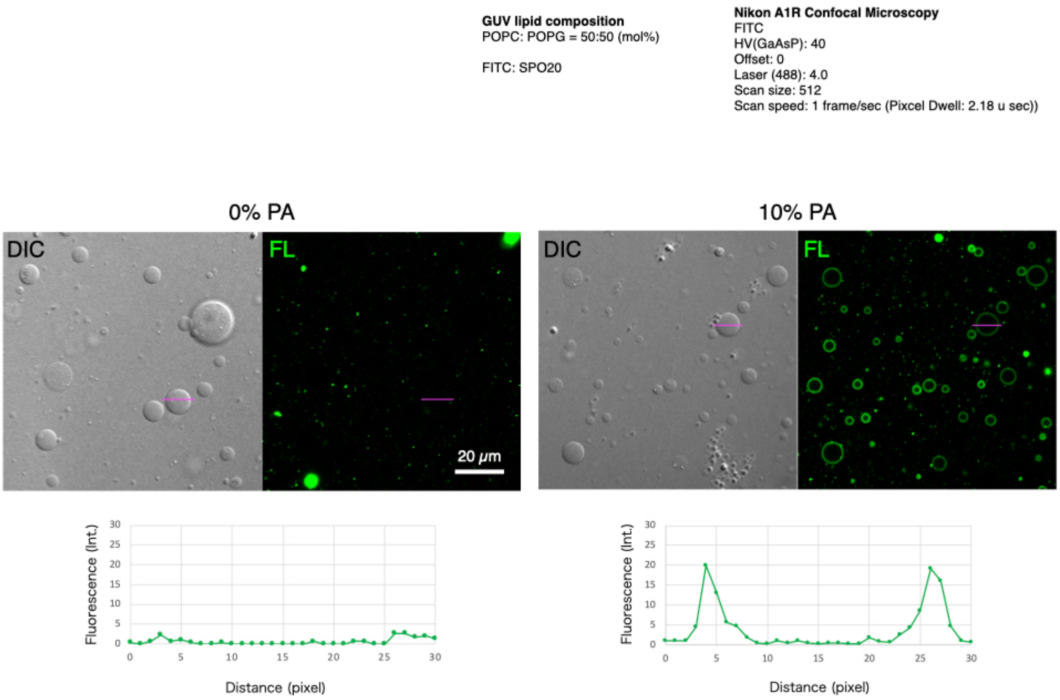

Supplementary Figure 19. Phosphatidic acid (PA)-specific membrane binding of GFP-Spo. A fluorescent probe, GFP-SPO, was introduced outside of GUVs which contain 10 % or 0% PA in the lipid composition. GUVs were observed with differential interference contrast (DIC) and 488 laser (FL). The fluorescent intensity on the given red lines are shown at the bottom of the images. Parameters in confocal microscopy are shown above the images.

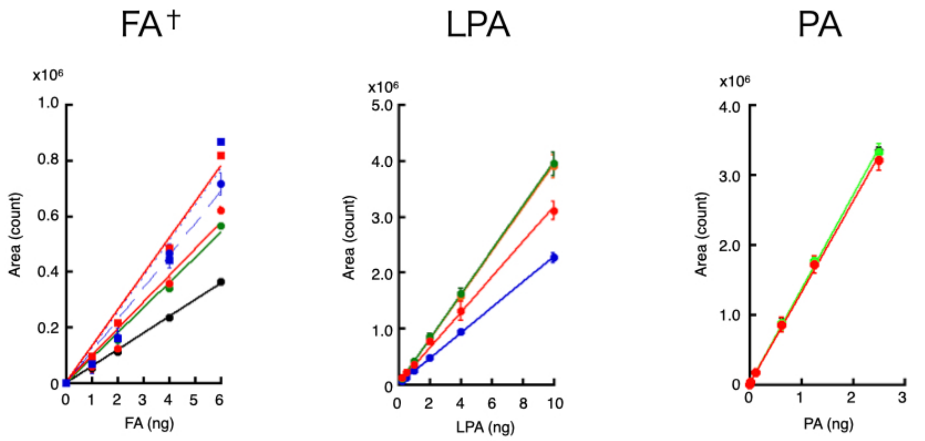

FA	Slope	R^2
12:0	6.0×10^4	0.99
14:0	8.0×10^4	0.99
16:1	1.0×10^5	0.99
16:0	1.0×10^5	0.96
18:1	1.0×10^5	0.95
18:0	1.0×10^5	0.98

LPA	Slope	R^2
14:0	2.3×10^5	0.99
16:0	3.9×10^5	0.99
18:1	4.0×10^5	0.99
18:0	3.2×10^5	0.99

PA	Slope	R^2
16:0/16:0	1.0×10^6	0.99
16:0/18:1	1.0×10^6	0.99
18:1/18:1	1.0×10^6	0.99

Supplementary Figure 20. Calibration curves of the standard fatty acids (FA), lysophosphatidic acids (LPA), and phosphatidic acids (PAs), for the quantification of cell-free synthesized lipids. The type of lipids, slope, and R^2 are shown at the bottom of the graphs. Error bars indicate standard deviation of triplicate measurements. † Each time the samples were analyzed by LC/MS, the standard fatty acids were analyzed at the same time.

Supplementary Table 1. Phospholipid concentration to be synthesized internally required to double the surface area of the vesicle.

Vesicle diameter (μm)	Number of phospholipids on vesicle surface	Internal volume (nL)	Necessary conc. of de novo phospholipids (mM)
10	1E+09	5E-04	3.0
20	4E+09	4E-03	1.5
30	9E+09	1E-02	1.0
40	2E+10	3E-02	0.8
50	2E+10	7E-02	0.6
60	3E+10	1E-01	0.5

Supplementary Table 2. Reaction composition for cell-free synthesis.

- Standard PURE system (Just for protein synthesis)

Stock conc.		component	Vol. (μL)	Final conc.	
		PURE*frex*2.0 Sol. I	10		
		PURE*frex*2.0 Sol. II	1		
		PURE*frex*2.0 Sol. III	2		
		DnaKJE mix	1		
2000	mM	Sucrose	2	200	mM
100	nM	DNAs for PlsX, PlsY, and PlsC	1	5	nM each
40	mg/mL	Liposomes	1	20	mg/mL
		MilliQ	2		
		sum	20		

- For LPA/PA synthesis

Stock conc.		component	Vol. (μL)	Final conc.	
		PURE*frex*2.0 Sol. I (custom)	6		
		PURE*frex*2.0 Sol. II	1		
		PURE*frex*2.0 Sol. III	2		
		DnaKJE mix	1		
2000	mM	Sucrose	2	200	mM
100	nM	DNAs for PlsX, PlsY, and PlsC	1	5	nM each
40	mg/mL	Liposomes	1	20	mg/mL
		FA mix	2		
50	mM	G3P	0.5	1.25	mM
		MilliQ	3.5		
		sum	20		

- For PA synthesis with CoA recycling

Stock conc.		component	Vol. (μL)	Final conc.	
		PURE*frex*2.0 Sol. I (custom)	6		
		PURE*frex*2.0 Sol. II	1		
		PURE*frex*2.0 Sol. III	2		
		DnaKJE mix	1		
2000	mM	Sucrose	2	200	mM
100	nM	DNAs for PlsX, PlsY, and PlsC	1	5	nM each
40	mg/mL	Liposomes	1	20	mg/mL

		FA mix	2		
50	mM	G3P	0.5	1.25	mM
400	μM	ACS	0.5	10.0	μM
400	μM	AccBC	0.5	10.0	μM
400	μM	AccDA	0.5	10.0	μM
		MilliQ	3.5		
		sum	20		

- For FA mix

Stock conc. (μM)	component	Vol. (μL)	Final conc. (μM)	×50 reaction
1000	FabA	0.2	10	10
1000	FabB	0.2	10	10
100	FabD	0.2	1	10
100	FabF	0.2	1	10
100	FabG	0.2	1	10
100	FabH	0.2	1	10
1000	FabI	0.2	10	10
1000	ACP	0.6*	30	30*
33.3	FabZ		1	
	sum	2.0		100#

*As a mixture of ACS and FabZ.

#This mixture was aliquoted in 2-5 μl before store in a freezer to avoid repeating freeze-and-thaw.

- Lipid synthesis reaction

Stock conc. (mM)	Component	Vol. (μL)	Final conc. (mM)
	Cell-Free mix	20	
100	NADPH	0.5	2
100	NADH	0.5	2
100	Acetyl-CoA	0.5	2
100	Malonyl-CoA	1	4
100	Potassium phosphate (pH 7.4)	2.5	10

	sum	25	

- For PA synthesis inside GUVs

Stock conc.		component	Vol. (μL)	Final conc.	
		PURE*frex*2.0 Sol. I (custom)	6		
		PURE*frex*2.0 Sol. II	1		
		PURE*frex*2.0 Sol. III	2		
		DnaKJE mix	1		
2000	mM	Sucrose	2	200	mM
33.3	nM	DNAs for PlsX, PlsY, and PlsC	3	5	nM each
		FA mix	2		
50	mM	G3P	0.5	1.25	mM
400	μM	ACS	0.5	10.0	μM
400	μM	AccBC	0.5	10.0	μM
200	μM	AccDA	0.5	5.0	μM
40	mM	CoA	0.5	1	mM
50	mM	ATP	0.5	1.25	mM
		sum	20		

The mixture was mixed with 12 % (w/v) Ficoll PM70.

Supplementary Table 3. DNA constructs for protein purification.

Enzyme	Plasmid	Host cell	Antibiotic	HisTag [N/C]	M. W. [kDa]
FabA*	pXY-FabA	BL21 (DE3)	Kanamycin	N	18.9
FabB*	pXY-FabB	BL21 (DE3)	Kanamycin	N	42.6
FabD*	pXY-FabD	BL21 (DE3)	Kanamycin	N	32.4
FabF*	pXY-FabF	BL21 (DE3)	Kanamycin	N	43.0
FabG*	pXY-FabG	BL21 (DE3)	Kanamycin	N	25.6
FabH*	pXY-FabH	BL21 (DE3)	Kanamycin	N	33.5
FabI*	pXY-FabI	BL21 (DE3)	Kanamycin	N	27.9
FabZ*	pXY-FabZ	C43(DE3)	Kanamycin	N	17.0
TesA*	pTL30	BL21 (DE3)	Kanamycin	N†	20.5
ACP*	pTL14§	BL21 (DE3)	Kanamycin	N and C	8.6
SFP*	pCDF-sfp§	BL21 (DE3)	Streptomycin	non	26.0
ACS	pET28a-acs	C43 (DE3)	Kanamycin	N	75.3
AccBC	pET28a-accBC$	C43 (DE3)	Kanamycin	N (of *accB*)	16.7 (*accB*)<br>49.3 (*accC*)
BirA	pCDF-birA$	C43 (DE3)	Streptomycin	non	35.3
AccDA	pACS275**	C43 (DE3)	Kanamycin	C (of *accD*)	35.2 (*accA*)<br>33.3 (*accD*)

*see Yu *et al.* 2011 PNAS[1], †without the leader sequence, §pTL14 and pCDF-sfp were co-transformed in cells to express holo-ACP, $pET28a-accBC and pCDF-birA were co-transformed in cells to biotinylate AccB, **see Smith *et al.* 2014 J. Bacteriol. [2].

Abbreviations: Fab, fatty acid binding protein; TesA, thioesterase I; ACP, acyl carrier protein; SFP, 4'-phosphopantetheinyl transferase; ACS, acetyl-CoA synthetase; Acc, acetyl-CoA carboxylase; BirA, biotin-[acetyl-CoA-carboxylase] ligase.

Supplementary Table 4. DNA sequences of genes used in this study. The regions of open reading frame are shown as lowercases, and initial or stop codons were shown as boldfaces.

**For in vitro expression**
plsX(*E. coli*)<br>GAAATTAATACGACTCACTATAGGGAGACCACAACGGTTTCCCTCTAGAA<br>ATAATTTTGTTTAACTTTAAGAAGGAGATATACC**atg**acacgtttaactttagcgttag<br>atgtcatgggaggggattttggcccttccgtgacagtgcctgcagcattgcaggcactgaattctaattcgca<br>actcactcttcttttagtcggcaattccgacgccatcacgccattacttgctaaagctgactttgaacaacgttc<br>gcgtctgcagattattcctgcgcagtcagttatcgccagtgatgcccggccttcgcaagctatccgcgccagt<br>cgtgggagttcaatgcgcgtggccctggagctggtgaaagaaggtcgagcgcaagcctgtgtcagtgccg<br>gtaataccggggcgctgatggggctggcaaaattattactcaagcccctggaggggattgagcgtccggc<br>gctggtgacggtattaccacatcagcaaaagggcaaaacggtggtccttgacttaggggccaacgtcgatt<br>gtgacagcacaatgctggtgcaatttgccattatgggctcagttctggctgaagaggtggtggaaattccca<br>atcctcgcgtggcgttgctcaatattggtgaagaagaagtaaagggtctcgacagtattcgggatgcctcag<br>cggtgcttaaaacaatcccttctatcaattatatcggctatcttgaagccaatgagttgttaactggcaagaca<br>gatgtgctggtttgtgacggctttacaggaaatgtcacattaaagacgatggaaggtgttgtcaggatgttcctt<br>tctctgctgaaatctcagggtgaagggaaaaaacggtcgtggtggctactgttattaaagcgttggctacaa<br>aagagcctgacgaggcgattcagtcacctcaaccccgaccagtataacggcgcctgtctgttaggattgcg<br>cggcacggtgataaaaagtcatggtgcagccaatcagcgagcttttgcggtcgcgattgaacaggcagtg<br>caggcggtgcagcgacaagttcctcagcgaattgccgctcgcctggaatctgtatacccagctggttttgag<br>ctgctggacggtggcaaaagcggaactctgcgg**tag**CAAAGCCCGAAAGGAAGCTGAGT<br>TGGCTGCTGCCACCGCTGAGCAATAACTAGC
plsY(*E. coli*)<br>GAAATTAATACGACTCACTATAGGGAGACCACAACGGTTTCCCTCTAGAA<br>ATAATTTTGTTTAACTTTAAGAAGGAGATATACC**atg**tcggcaatagcgcctggtatg<br>attttaatcgcttatctttgtggttctatctcgagtgctattttggtgtgccgcttatgtgggcttccagacccacgca<br>ctagtggatcagggaaccctggagctaccaacgttttgcggataggtggaaaggggcagcggtggctgt<br>actgatttttgatgtcttgaagggaatgttgcccgtctggggagcgtatgaactgggtgtcagtcctttctggctg<br>ggattgatcgctattgcggcctgcttgggccacatctggccggttttctttgggtttaagggtggaaagggcgtc<br>gctactgcatttggcgcgatagcccccatagggtgggacctgaccggagttatggccggtacttggttgctg<br>accgtgcttcttagcgggtattcgagcttaggcgcaattgtgagtgctttgattgccccgttttacgtctggtggtt<br>caaaccacaatttacgttcccagtatctatgctgtcttgtttgatattgttgcgccaccatgataacatccagcgc<br>ctttggagaagacaggagactaagatttggacaaaattcaaacgcaaacgtgaaaaagacccagag**ta**<br>**a**CAAAGCCCGAAA
plsC(*E. coli*)

TAATACGACTCACTATAGGGAGACCACAACGGTTTCCCTCTAGAAATAAT<br>TTTGTTTAACTTTAAGAAGGAGATATACC**atg**ctatatatctttcgtcttattattaccgtgatt<br>tacagcatcttagtctgtgtattcggctccatttactgccttttcagcccgcgtaacccgaaacatgtggccacc<br>tttgggcatatgtttggccgtcttgcgccgctgtttggcctgaaagttgagtgccgtaaacctacagacgctga<br>aagctacggcaatgctatctatatcgctaaccaccagaacaactatgacatggtgacagcatcgaacatc<br>gtgcaaccgccgacggtgacggtaggtaaaaagagcttgctgtggatccccttcttcgggcagttgtactgg<br>ttaaccggcaacttattgatcgacagaaacaatcgcactaaagctcacggcaccattgcggaagtagtga<br>atcacttcaaaaaacgccgtatttccatctggatgttcccggaaggaacccgcagccgtggtcgcggcctg<br>ctaccgttcaagactggagcatttcacgcggcaattgcggcgggcgtcccgattattcccgtgtgcgtctcta<br>caacttcgaataagattaatcttaatcgactgcacaacggtctggtgattgtcgaaatgctgccgccaattga<br>cgtcagtcagtatggcaaagatcaggttcgtgagctggctgcccattgtcgttcgataatggaacaaaaaat<br>cgccgagctcgataaagaagtcgcagaacgcgaagccgccggaaaagtt**taa**
plsX(*E. coli*)-6His<br>GAAATTAATACGACTCACTATAGGGAGACCACAACGGTTTCCCTCTAGAA<br>ATAATTTTGTTTAACTTTAAGAAGGAGATATACC**atg**acacgtttaactttagcgttag<br>atgtcatgggaggggattttggcccttccgtgacagtgcctgcagcattgcaggcactgaattctaattcgca<br>actcactcttcttttagtcggcaattccgacgccatcacgccattacttgctaaagctgactttgaacaacgttc<br>gcgtctgcagattattcctgcgcagtcagttatcgccagtgatgcccggccttcgcaagctatccgcgccagt<br>cgtgggagttcaatgcgcgtggccctggagctggtgaaagaaggtcgagcgcaagcctgtgtcagtgccg<br>gtaataccggggcgctgatggggctggcaaaattattactcaagcccctggaggggattgagcgtccggc<br>gctggtgacggtattaccacatcagcaaaagggcaaaacggtggtccttgacttaggggccaacgtcgatt<br>gtgacagcacaatgctggtgcaatttgccattatgggctcagttctggctgaagaggtggtggaaattccca<br>atcctcgcgtggcgttgctcaatattggtgaagaagaagtaaagggtctcgacagtattcgggatgcctcag<br>cggtgcttaaaacaatcccttctatcaattatatcggctatcttgaagccaatgagttgttaactggcaagaca<br>gatgtgctggtttgtgacggctttacaggaaatgtcacattaaagacgatggaaggtgttgtcaggatgttcctt<br>tctctgctgaaatctcagggtgaagggaaaaaacggtcgtggtggctactgttattaaagcgttggctacaa<br>aagagcctgacgaggcgattcagtcacctcaaccccgaccagtataacggcgcctgtctgttaggattgcg<br>cggcacggtgataaaaagtcatggtgcagccaatcagcgagcttttgcggtcgcgattgaacaggcagtg<br>caggcggtgcagcgacaagttcctcagcgaattgccgctcgcctggaatctgtatacccagctggttttgag<br>ctgctggacggtggcaaaagcggaactctgcggcatcatcatcatcatcat**taa**ATCCGGCTGC
plsY(*E. coli*)-6His<br>GAAATTAATACGACTCACTATAGGGAGACCACAACGGTTTCCCTCTAGAA<br>ATAATTTTGTTTAACTTTAAGAAGGAGATATACC**atg**tcggcaatagcgcctggtatg<br>attttaatcgcttatctttgtggttctatctcgagtgctattttggtgtgccgcttatgtgggcttccagacccacgca<br>ctagtggatcagggaaccctggagctaccaacgttttgcggataggtggaaaaggggcagcggtggctgt<br>actgatttttgatgtcttgaagggaatgttgcccgtctggggagcgtatgaactgggtgtcagtcctttctggctg

ggattgatcgctattgcggcctgcttgggccacatctggccggttttctttgggtttaagggtggaaagggcgtc<br>gctactgcatttggcgcgatagcccccatagggtgggacctgaccggagttatggccggtacttggttgctg<br>accgtgcttcttagcgggtattcgagcttaggcgcaattgtgagtgctttgattgccccgttttacgtctggtggtt<br>caaaccacaatttacgttcccagtatctatgctgtcttgtttgatattgttgcgccaccatgataacatccagcgc<br>ctttggagaagacaggagactaagatttggacaaaattcaaacgcaaacgtgaaaaagacccagagca<br>tcatcatcatcatcat**taa**ATCCGGGCTGC
plsY(*E. coli*)-6His<br>GAAATTAATACGACTCACTATAGGGAGACCACAACGGTTTCCCTCTAGAA<br>ATAATTTTGTTTAACTTTAAGAAGGAGATATACC**atg**tcggcaatagcgcctggtatg<br>attttaatcgcttatctttgtggttctatctcgagtgctattttggtgtgccgcttatgtgggcttccagacccacgca<br>ctagtggatcagggaaccctggagctaccaacgttttgcggataggtggaaaaggggcagcggtggctgt<br>actgatttttgatgtcttgaagggaatgttgcccgtctggggagcgtatgaactgggtgtcagtcctttctggctg<br>ggattgatcgctattgcggcctgcttgggccacatctggccggttttctttgggtttaagggtggaaagggcgtc<br>gctactgcatttggcgcgatagcccccatagggtgggacctgaccggagttatggccggtacttggttgctg<br>accgtgcttcttagcgggtattcgagcttaggcgcaattgtgagtgctttgattgccccgttttacgtctggtggtt<br>caaaccacaatttacgttcccagtatctatgctgtcttgtttgatattgttgcgccaccatgataacatccagcgc<br>ctttggagaagacaggagactaagatttggacaaaattcaaacgcaaacgtgaaaaagacccagagca<br>tcatcatcatcatcat**taa**ATCCGGGCTGC
plsY(*E. coli*)-6His<br>GAAATTAATACGACTCACTATAGGGAGACCACAACGGTTTCCCTCTAGAA<br>ATAATTTTGTTTAACTTTAAGAAGGAGATATACC**atg**tcggcaatagcgcctggtatg<br>attttaatcgcttatctttgtggttctatctcgagtgctattttggtgtgccgcttatgtgggcttccagacccacgca<br>ctagtggatcagggaaccctggagctaccaacgttttgcggataggtggaaaaggggcagcggtggctgt<br>actgatttttgatgtcttgaagggaatgttgcccgtctggggagcgtatgaactgggtgtcagtcctttctggctg<br>ggattgatcgctattgcggcctgcttgggccacatctggccggttttctttgggtttaagggtggaaagggcgtc<br>gctactgcatttggcgcgatagcccccatagggtgggacctgaccggagttatggccggtacttggttgctg<br>accgtgcttcttagcgggtattcgagcttaggcgcaattgtgagtgctttgattgccccgttttacgtctggtggtt<br>caaaccacaatttacgttcccagtatctatgctgtcttgtttgatattgttgcgccaccatgataacatccagcgc<br>ctttggagaagacaggagactaagatttggacaaaattcaaacgcaaacgtgaaaaagacccagagca<br>tcatcatcatcatcat**taa**ATCCGGGCTGC
plsY(*E. coli*)-6His<br>GAAATTAATACGACTCACTATAGGGAGACCACAACGGTTTCCCTCTAGAA<br>ATAATTTTGTTTAACTTTAAGAAGGAGATATACC**atg**tcggcaatagcgcctggtatg<br>attttaatcgcttatctttgtggttctatctcgagtgctattttggtgtgccgcttatgtgggcttccagacccacgca<br>ctagtggatcagggaaccctggagctaccaacgttttgcggataggtggaaaaggggcagcggtggctgt<br>actgatttttgatgtcttgaagggaatgttgcccgtctggggagcgtatgaactgggtgtcagtcctttctggctg

ggattgatcgctattgcggcctgcttgggccacatctggccggttttctttgggtttaagggtggaaagggcgtcgctactgcatttggcgcgatagcccccatagggtgggacctgaccggagttatggccggtacttggttgctgaccgtgcttcttagcgggtattcgagcttaggcgcaattgtgagtgctttgattgccccgttttacgtctggtggttcaaaccacaatttacgttcccagtatctatgctgtcttgtttgatattgttgcgccaccatgataacatccagcgcctttggagaagacaggagactaagatttggacaaaattcaaacgcaaacgtgaaaaagacccagagcatcatcatcatcatcat**taa**ATCCGGGCTGC
plsC(*E. coli*)-6His<br>TAATACGACTCACTATAGGGGAATTGTGAGCGGATAACAATTCCCCTCTAGAAATAATTTTGTTTAACTTTAAGAAGGAGATATACC**atg**ctatatatctttcgtcttattattaccgtgatttacagcatcttagtctgtgtattcggctccatttactgccttttcagcccgcgtaacccgaaacatgtggccacctttgggcatatgtttggccgtcttgcgccgctgtttggcctgaaagttgagtgccgtaaacctacagacgctgaaagctacggcaatgctatctatatcgctaaccaccagaacaactatgacatggtgacagcatcgaacatcgtgcaaccgccgacggtgacggtaggtaaaaagagcttgctgtggatccccttcttcgggcagttgtactggttaaccggcaacttattgatcgacagaaacaatcgcactaaagctcacggcaccattgcggaagtagtgaatcacttcaaaaaacgccgtatttccatctggatgttcccggaaggaacccgcagccgtggtcgcggcctgctaccgttcaagactggagcatttcacgcggcaattgcggcgggcgtcccgattattcccgtgtgcgtctctacaacttcgaataagattaatcttaatcgactgcacaacggtctggtgattgtcgaaatgctgccgccaattgacgtcagtcagtatggcaaagatcaggttcgtgagctggctgcccattgtcgttcgataatggaacaaaaaaatcgccgagctcgataaagaagtcgcagaacgcgaagccgccggaaaagttcatcatcatcatcatcat**taa**CGAT
sfgfp-plsX$_{wt}$(*E. coli*)<br>…TAATACGACTCACTATAGGGGAATTGTGAGCGGATAACAATTCCCCTCTAGAAATAATTTTGTTTAACTTTAAGAAGGAGATATACC**atg**agtaaaggagaagaacttttcactggagttgtcccaattcttgttgaattagatggtgatgttaatgggcacaaattttctgtccgtggagagggtgaaggtgatgcaacaaacggaaaacttacccttaaatttatttgcactactggaaaactacctgttccatggccaacacttgtcactactttaacttatggtgttcaatgcttttcccgttatccggatcacatgaaacggcatgactttttcaagagtgccatgcccgaaggttatgtacaggaacgcactatatctttcaaagatgacgggacctacaagacgcgtgctgaagtcaagtttgaaggtgatacccttgttaatcgtatcgagttaaaaggtattgattttaaagaagatggaaacattctcggacacaaactcgagtacaactttaactcacacaatgtatacatcacggcagacaaacaaaagaatggaatcaaagctaacttcaaaattcgccacaacgttgaagatggatccgttcaactagcagaccattatcaacaaaatactccaattggcgatggccctgtccttttaccagacaaccattacctgtcgacacaatctgtcctttcgaaagatcccaacgaaaagcgtgaccacatggtccttcttgagtttgtaactgctgctgggattacacatggcatggatgagctctacaaatcaggaagcggctcaggatccatgacacgtctaaccctggcgttagatgtcatgggaggggattttggcccttccgtgacagtgcctgcagcattgcaggcactgaattctaattcgcaactcactcttcttttagtcggcaattccgacgccatcacgccattacttgctaaagctgactttgaacaacgttcgcgtctgcagattattcctgcgcagtcagttatcgccagtgatgcccggccttcgcaag

ctatccgcgccagtcgtgggagttcaatgcgcgtggccctggagctggtgaaagaaggtcgagcgcaag
cctgtgtcagtgccggtaataccggggcgctgatggggctggcaaaattattactcaagcccctggagggg
attgagcgtccggcgctggtgacggtattaccacatcagcaaaagggcaaaacggtggtccttgacttagg
ggccaacgtcgattgtgacagcacaatgctggtgcaatttgccattatgggctcagttctggctgaagaggtg
gtggaaattcccaatcctcgcgtggcgttgctcaatattggtgaagaagaagtaaagggtctcgacagtatt
cgggatgcctcagcggtgcttaaaacaatcccttctatcaattatatcggctatcttgaagccaatgagttgtta
actggcaagacagatgtgctggtttgtgacggctttacaggaaatgtcacattaaagacgatggaaggtgtt
gtcaggatgttcctttctctgctgaaatctcagggtgaagggaaaaaacggtcgtggtggctactgttattaaa
gcgttggctacaaaagagcctgacgaggcgattcagtcacctcaaccccgaccagtataacggcgcctgt
ctgttaggattgcgcggcacggtgataaaaagtcatggtgcagccaatcagcgagcttttgcggtcgcgatt
gaacaggcagtgcaggcggtgcagcgacaagttcctcagcgaattgccgctcgcctggaatctgtatacc
cagctggttttgagctgctggacggtggcaaaagcggaactctgcgg**taa**CAAAGCCCGAAAGG
AAGCTGAGTTGGCTGCTGCCACCGCTGAGCAATAACTAGC…

plsY$_{wt}$(*E. coli*)-*sfgfp*
…TAATACGACTCACTATAGGGGAATTGTGAGCGGATAACAATTCCCCTC
TAGAAATAATTTTGTTTAACTTTAAGAAGGAGATATACC**atg**agtgcaatcgcgc
ctggaatgatcctcatcgcgtacctctgcggctccatttccagtgccattctggtttgccgcttgtgtgggctgcc
cgatccgcgaaccagcggctccggcaatccaggcgcaaccaatgtgttacgtatcggtggcaagggagc
agccgtagcagtactgattttcgacgttctgaaaggaatgttgcccgtctggggcgcgtatgaattaggtgtc
agccccttctggctaggcttaattgccatcgccgcctgtcttggacacatctggcccgttttcttcggatttaaag
gaggaaaaggcgttgctaccgcttttggtgccatcgcacccattggctgggatctcaccggagtaatggcg
ggaacctggttactgaccgtgctattgagcggatactcgtcgctgggagcgattgtcagtgcactgattgctc
cgttttatgtctggtggtttaagccacaattcaccttcccggtttcgatgctctcttgcctgatcctgctgcgtcatc
atgacaacatccaacgtctgtggcgtcgtcaggagacaaaaatctggacgaaattcaaaagaaagcgcg
aaaaggatcccgagtccggatcaggcagcggatcaatgagtaaaggagaagaacttttcactggagttgt
cccaattcttgttgaattagatggtgatgttaatgggcacaaattttctgtccgtggagagggtgaaggtgatgc
aacaaacggaaaacttacccttaaatttatttgcactactggaaaactacctgttccatggccaacacttgtc
actactttaacttatggtgttcaatgcttttcccgttatccggatcacatgaaacggcatgactttttcaagagtgc
catgcccgaaggttatgtacaggaacgcactatatctttcaaagatgacgggacctacaagacgcgtgctg
aagtcaagtttgaaggtgatacccttgttaatcgtatcgagttaaaaggtattgattttaaagaagatggaaac
attctcggacacaaactcgagtacaactttaactcacacaatgtatacatcacggcagacaaacaaaaga
atggaatcaaagctaacttcaaaattcgccacaacgttgaagatggatccgttcaactagcagaccattatc
aacaaaatactccaattggcgatggccctgtccttttaccagacaaccattacctgtcgacacaatctgtcctt
tcgaaagatcccaacgaaaagcgtgaccacatggtccttcttgagtttgtaactgctgctgggattacacatg
gcatggatgagctctacaaa**taa**CAAAGCCCGAAAGGAAGCTGAGTTGGCTGCTG
CCACCGCTGAGCAATAACTAGC…

sfgfp<br>**atg**agtaaaggagaagaacttttcactggagttgtcccaattcttgttgaattagatggtgatgttaatgggca<br>caaattttctgtccgtggagagggtgaaggtgatgcaacaaacggaaaacttacccttaaatttatttgcact<br>actggaaaactacctgttccatggccaacacttgtcactactttaacttatggtgttcaatgcttttcccgttatcc<br>ggatcacatgaaacggcatgactttttcaagagtgccatgcccgaaggttatgtacaggaacgcactatatc<br>tttcaaagatgacgggacctacaagacgcgtgctgaagtcaagtttgaaggtgatacccttgttaatcgtatc<br>gagttaaaaggtattgattttaaagaagatggaaacattctcggacacaaactcgagtacaactttaactca<br>cacaatgtatacatcacggcagacaaacaaaagaatggaatcaaagctaacttcaaaattcgccacaac<br>gttgaagatggatccgttcaactagcagaccattatcaacaaaatactccaattggcgatggccctgtcctttt<br>accagacaaccattacctgtcgacacaatctgtcctttcgaaagatcccaacgaaaagcgtgaccacatg<br>gtccttcttgagtttgtaactgctgctgggattacacatggcatggatgagctctacaaa**taa**
**For protein purification**
sfc<br>**atg**aaaatctatggcatctacatggatcgtcctcttagccaggaagagaatgaacgctttatgagctttatctc<br>gccggagaaacaggagaaatgtcgccgtttctatcacaaggaagatgcacaccgtaccttattgggtgac<br>gttctggttcgttcggtgattagccgccaatatcagctggacaaagcggacattcgctttagtgctcaggaata<br>tggtaaaccgtgcattccggatctcccagacgcgcactttaacattagccattcaggccggtgggtcatttgc<br>gcatttgacagccatccgatcggtatcgatattgagaagatgaaaccgatttctctggaaattgccaaacgct<br>tcttttccaaaactgaatattctgatctgctggcgaagaacaaagatgaacagaccgattacttctaccatctg<br>tggtccatgaaagaatcgttcattaagcaggaagggaaaggactctctctgcctttagactcatttagtgtgc<br>gcctgcaccaagatggccaagtgagcatcgaactgccggattcccatacaccgtgctatatcaaaacgta<br>cgaagtcgaccccggctacaaaatggccgtatgtgctgcccatccagatttccccgaagatattaccatgtt<br>gagttatgaggcgcttctg**taa**
acs<br>**atg**ggcagcagccatcatcatcatcatcacagcagcggcctggtgccgcgcggcagccatatgagccaa<br>atccataagcataccattccagcgaacattgctgaccgctgcttaatcaatccgcagcaatatgaggcgatg<br>tatcaacagagcatcaacgtccccgatacattctggggtgaacagggaaagattttagactggatcaaacc<br>gtatcagaaggtgaagaacacgtcatttgcaccaggaaacgtctctattaagtggtatgaagacggaca<br>ctgaaccttgcagccaattgtctggaccgtcacctccaagagaacggcgatcgcactgcgatcatctggga<br>aggtgacgatgcgtcccagtcgaagcacatctcttacaaggaactgcaccgtgatgtttgtcgtttcgccaac<br>actctgttggaacttggtatcaagaaaggggatgttgtcgccatttatatgcccatggttccggaagctgcagt<br>tgcaatgcttgcctgcgcgcgcatcggtgcagttcacagtgtgatcttcggtggcttcagccctgaggcggta<br>gctggccgcatcatcgactctaattctcgcctcgtaattacgagtgacgaaggagtccgcgctggtcgctcta<br>ttcctttgaagaagaatgttgacgatgccttaaagaacccgaacgttacgagcgtcgagcacgtggtggtac<br>tgaaacgcaccggcggtaagatcgactggcaggaaggacgcgatttgtggtggcacgatctcgtcgaac<br>aggcttcggaccaacaccaggctgaggagatgaacgcggaagatccgctgtttatcctgtacactagtgg

atcgaccggcaagcctaagggtgtgttacacacaacgggtggttatttagtgtatgcagcgttaaccttcaaa
tatgtatttgattatcatcccggcgatatctactggtgcacagcagatgtcgggtgggttacggggcattcatat
ttgctgtatggtccgttagcctgtggtgcgacaacgctgatgttcgaaggagttccaaactggccaacgcca
gcgcgcatggctcaggtcgttgataaacatcaggtcaatatcctttataccgcaccgaccgccatccgtgca
ttgatggccgaaggtgacaaggctattgaaggcacagaccgttctagtttgcgcatccttggtagcgttggcg
aaccaatcaaccccgaagcttgggagtggtactggaagaagattggtaatgaaaagtgcccggttgtaga
cacatggtggcagaccgagacgggaggcttcatgattactcccctcccaggcgcgaccgaattgaaggc
gggatcggcgacccgtccgttctttggcgtgcagccagccttagttgataacgaaggtaacccactggagg
gagctacggaaggatctttagtcatcaccgattcttggccgggccaagctcgcactcttttcggcgaccatga
acgcttcgagcagacttatttcagcacattcaagaacatgtacttttcaggtgatggcgcgcgccgtgacga
agacggttattattggatcacgggccgtgtagacgatgtgctgaacgtctctgggcatcgcttaggcaccgc
ggagatcgaatcagctcttgtagcccatcctaagattgcagaagccgcagtagtgggtatccctcataatat
caaaggccaggcgatttacgcttatgttaccttaaatcacggtgaagagcccagcccggaactgtatgcgg
aagttcgcaactgggtgcgcaaggaaattggaccgttggccaccccagacgttctgcactggaccgattcg
ttgccgaagactcgctcgggcaaaattatgcgccgcatcttacgcaagattgcagccggcgacacttcaaa
cttaggtgacacttctaccttagctgacccgggagtggttgaaaagttgttggaggagaaacaggccatcgc
aatgccctccctcgagcaccaccaccaccaccac**tga**

accBC

atggacattcgcaaaattaagaaattgattgaattagttgaagagtcgggaatttctgaactcgaaatctcag
agggcgaggagagcgtccgcatcagtcgtgcagcaccggctgcatcttttccggtgatgcagcaagcgta
cgcggccccaatgatgcagcaaccagcacaaagcaatgccgcggccccggctaccgtcccctcgatgg
aagccccggcggcggcggaaattagcggccatattgtgcgcagcccgatggtggggacattctatcgcac
ccctagtccggacgctaaagctttcatcgaagtgggtcagaaggtgaacgttggtgataccctgtgcattgtt
gaagcgatgaaaatgatgaaccaaatcgaggccgataaatccggcactgtaaaagccattctggtagaa
tccggtcagccggttgaatttgacgaacctctggttgtgatcgaa**taa**ggaggtggatccg**atg**cttgataag
atcgtcattgctaaccgtggggaaattgcgctccgcatccttcgcgcatgcaaagagttgggcatcaagacc
gtcgccgttcactctagtgcggatcgtgacctgaagcacgttttgttggcagacgaaaccgtctgtattggccc
ggcacccagtgtgaagtcgtatctgaacatcccggctattatttccgctgcggagatcaccggcgcagtagc
cattcatccgggctatggcttcctgtcagagaacgctaatttcgcagaacaggttgaacgcagcggattcat
cttcatcggtccgaaggccgaaactatccgcctgatgggcgataaggtgtccgccattgctgcgatgaaga
aggcgggtgtaccttgtgtccccggctccgatgggccgcttggcgacgatatggataagaaccgcgctatt
gccaaacgtattggttatccagtaattatcaaggccagcggcggtggtggcggtcgcggcatgcgtgtcgtt
cgcggtgacgccgaattggcgcaatcaattagtatgactcgcgcagaagcgaaggcggcttttagtaacg
atatggtttacatggaaaagtacttagaaaatccccgccatgttgagattcaggttttggcggacggtcaagg
taacgcgatttacctggccgaacgcgactgttccatgcaacgccgtcaccagaaggtggtagaagaagct
ccggcgccggggattactccagaactgcgccgctacattggagaacgctgcgcaaaagcgtgcgtggac

atcgggtaccgtggtgccggcacttttgaattcctcttcgaaaatggtgaattctacttcattgaaatgaatactc<br>gtatccaggtcgagcatcccgtaacagaaatgatcaccggtgttgatctgatcaaggaacagttacgcattg<br>cggcgggacagccactctctatcaaacaagaagaggtccatgtacgcggtcatgcagtggaatgtcgtat<br>caacgcagaagatccgaacacatttctcccatcacccggtaagattacgcgttttcacgcgccgggcggct<br>tcggagtccgttgggagtcacacatctacgcgggttacacagttccaccgtactacgacagtatgattggaa<br>agttaatttgctatggagaaaaccgcgatgtggccatcgctcgtatgaagaatgccttgcaagaacttatcat<br>tgatggcatcaagaccaacgttgatcttcaaattcgtatcatgaacgacgagaacttccaacacggtggaa<br>caaacattcattacttagagaagaaattgggcctgcaagaaaag**taa**
birA<br>**atg**ggcaaggacaatacggtgcccctcaaacttattgcattactcgctaatggggagtttcatagcggcga<br>gcaattaggagagacccttggtatgtcacgtgcggcgattaacaaacacattcagaccctgcgcgattggg<br>gagtcgatgtgttcaccgtcccgggtaaaggttattcccttccggagccgatccaattgctcaatgcgaagca<br>gattctgggacagcttgatggcgggtcggtagccgtgctccctgttatcgatagcacgaaccagtatctgtta<br>gaccgtattggcgaactgaaatccggcgatgcctgcatcgcggaataccagcaggccgggcgtggtcgtc<br>gtggccgcaaatggttctctccatttggagccaatctgtatttgtcaatgttctggcgcctggaacaaggccca<br>gccgcagcaatcgggttaagcttggtcattggaatcgttatggcagaggttttgcgcaaactgggcgccgat<br>aaagttcgcgtcaaatggccgaatgatttgtacttacaggaccgtaagttggcagggatcctggtggaatta<br>actggtaagaccggtgacgctgcgcaaatcgtaatcggtgcggggatcaacatggcgatgcgccgcgtg<br>gaggaatcggtagttaatcagggctggatcactctgcaagaggcggggatcaacctggatcgcaacaca<br>ctggctgcgatgctgatccgtgaactgcgcgcggcactggagctgttcgaacaagaaggcctggctccgta<br>cctgagtcgctgggagaaactggacaacttcattaaccgtccggtaaaactgattattggcgacaaggaaa<br>tcttcggtatttctcgcgggattgacaaacaaggcgcactgctgctggaacaggacggtattattaagccttg<br>gatgggcggcgaaattagcctgcgcagcgccgagaaa**taa**
GFP-Spo<br>**atg**ggcagcagccatcatcatcatcatcacagcagcggcctggtgccgcgcggcagccatatgtctaaag<br>gggaagagctgttcaccggcgtagttcccattctcgtagagttggatggagatgtcaacggccacaaattca<br>gcgttcgcggagaaggagagggtgacgcaacgaacggcaaacttacgttaaagttcatctgtaccactgg<br>caaattaccggtcccttggcctacactggttacaaccttaacatacggtgtgcaatgttttagccgctatcctga<br>ccacatgaaacgccatgatttcttcaagtccgcgatgccggaaggttatgtccaagaacgtacgattagctt<br>caaagatgacggcacatataaaacccgcgctgaagtaaaatttgagggcgacactctcgtcaaccgcat<br>cgaactgaaaggaatcgattttaaagaagacggtaatattttgggtcataaacttgaatataactttaacagt<br>cataacgtttacattactgcggacaaacagaagaatgggatcaaggctaactttaaaatccgtcacaacgt<br>ggaggatggcagcgtgcagctggcagatcactatcagcaaaatacaccgattggggacggtccagtgct<br>gttacccgataatcattatctcagtacccagtctgtgctgtcaaaagaccccaatgaaaagcgcgaccacat<br>ggtgttactggaattcgtcactgctgcgggtattactcatggaatggatgaattgtacaaaggtggtggcggat<br>caggaggaggcggtagtatggacaattgctcgggatcacgccgtcgcgaccgtctgcacgtgaagttaaa

gagcctgcgtaacaaaattcataaacaactgcacccgaactgccgttttgatgatgcaacgaaaacatctc
tcgagcaccaccaccaccaccac**tga**

Supplementary Table 5. Primer sequence.

Primer name	Sequence	Base
sfp-fw	AGGAGATATACCATGAAAATCTATGGCATCTACAT	36
sfp-rv	CTCCCAATTGGGATCTTACAGAAGCGCCTCATAAC	35
PURE_pET_RV	GGTATATCTCCTTCTTAAAGTTAAACAAAATTATT	35
Pet-X/Y-FW	TAACAAAGCCCGAAAGGAAGCTGAGTTGGCTGCT	34
PURE_X_side_FW	AGAAGGAGATATACCATGACACGTCTAACCCTGGC	35
plsX_side_RV	TTTCGGGCTTTGTTACCGCAGAGTTCCGCTTTTGC	35
SG_sfGFP-FW	TCAGGCAGCGGATCAATGAGTAAAGGAGAAGAACT	35
SG_sfGFP-RV	TTTCGGGCTTTGTTATTTGTAGAGCTCATCCAT	33
plsY_side_FW	AGAAGGAGATATACCATGAGTGCAATCGCGCCTGG	35
plsY_side_RV	TTTCGGGCTTTGTTACTCGGGATCCTTTTCGCGCT	35
Y_vector35-RV	TGATCCGCTGCCTGATCCGGACTCGGGATCCTTTTC	36
sfGFP_SG-FW	AGAAGGAGATATACCATGAGTAAAGGAGAAGAACT	35
sfGFP_SG-RV	GGATCCTGAGCCGCTTCCTGATTTGTAGAGCTCATCCAT	39
X_vector68-FW	AGCGGCTCAGGATCCATGACACGTCTAACCCTGGC	35
Xmut_FW	CTTTAAGAAGGAGATATACCATGACACGTTTAACTTTAGC	40
Yopt-FW	AGAAGGAGATATACCATGTCGGCAATAGCGCCTGG	35
Yopt-RV	TTTCGGGCTTTGTTACTCTGGGTCTTTTTCACGTT	35
Yopt-His-RV	GCAGCCCGGATTTAATGATGATGATGATGATGCTCTGGGTCTTTT TCACGTT	52
Xmut_His_RV	GCAGCCGGATTTAATGATGATGATGATGATGCCGCAGAGTTCCG CTTTTGCC	52
PlsC_His_RV	ATCGTTAATGATGATGATGATGATGAACTTTTCCGGCGG	39
Universal primer	GAAATTAATACGACTCACTATAGGGAGACCACAACGGTTTCCCTC TAGAAATAATTTTGTTTAACTTTAAGAAGGAGATATACCA	85
T7p	TAATACGACTCACTATAGGG	20
T7t	GCTAGTTATTGCTCAGCGG	19

Supplementary Table 6. Buffer contents for protein purification.

Buffer	Contents
A	50 mM Tris-HCl (pH 7.6), 2 mM dithiothreitol (DTT), 10 % glycerol
B	50 mM Tris-HCl (pH 7.6), 2 mM dithiothreitol (DTT), 10 % glycerol, 1 M Imidazol
C	50 mM Tris-HCl (pH 7.6), 2 mM dithiothreitol (DTT), 10 % glycerol, 1 M NaCl
D	100 mM potassium phosphate (pH 7.4), 300 mM NaCl, 1 mM DTT
E	100 mM potassium phosphate (pH 7.4), 300 mM NaCl, 1 mM DTT, 1 M Imidazol
F	50 mM Tris-HCl (pH 8.0), 10 % glycerol
G	50 mM Tris-HCl (pH 8.0), 10 % glycerol, 1 M NaCl
H	20 mM potassium phosphate (pH 8.0), 50 mM NaCl, 2-mercaptoethanol, PI*
I	20 mM potassium phosphate (pH 8.0), 500 mM NaCl, 2-mercaptoethanol, PI*
J	50 mM Tris-HCl (pH 8.0), 100 mM NaCl, 10 % glycerol
K	50 mM Tris-HCl (pH 8.0), 1 M NaCl, 10 % glycerol
L	20 mM Tris-HCl (pH 8.0), 300 mM NaCl, 10 % glycerol
M	20 mM Tris-HCl (pH 8.0), 500 mM NaCl, 10 % glycerol
N	20 mM Tris-HCl (pH 8.0), 500 mM NaCl, 10 % glycerol, 1 M Imidazol
O	20 mM Tris-HCl (pH 8.0) and 10 % glycerol
P	20 mM Tris-HCl (pH 8.0) and 10 % glycerol, 1 M NaCl

*Protease inhibitor cocktail

Supplementary Table 7. Components for *in vitro* fatty acid synthesis reaction.

Component	Stock conc.	Used vol. (μL)	Final conc.
FabA	500 μM	0.4	10 μM
FabB	500 μM	0.4	10 μM
FabD	50 μM	0.4	1 μM
FabE	50 μM	0.4	1 μM
FabG	50 μM	0.4	1 μM
FabH	50 μM	0.4	1 μM
FabI	500 μM	0.4	10 μM
TesA	300 μM	2.0	30 μM
FabZ	20 μM*	1.0*	1 μM
ACP	600 μM*		30 μM
NADH	50 mM	1.0	2.5 mM
NADPH	50 mM	1.0	2.5 mM
^{13}C-Acetyl-CoA/Acetyl-CoA	10 mM	2.0	1 mM
^{13}C-Malonyl-CoA/Malonyl-CoA	25 mM	2.0	2.5 mM
Buffer†		8.2	
Total		20.0	

*As a mixture of ACP and FabZ

†100 mM sodium phosphate (pH 7.4), 300 mM NaCl, 1 mM DTT

Supplementary Table 8. The *m*/z value of analyzed lipids.

Lipid	Acyl chain 1	Acyl chain 2	m/z		^{13}C-labeled lipid	Acyl chain 1	Acyl chain 2	m/z
FA	C12:0		*199.1*		^{13}C-FA	C12:0		*211.9*
FA	C14:0		*227.1*		^{13}C-FA	C14:0		*241.2*
FA	C16:0		*255.1*		^{13}C-FA	C16:0		*271.2*
FA	C16:1		*253.1*		^{13}C-FA	C16:1		*269.2*
FA	C18:0		*283.2*		^{13}C-FA	C18:0		*301.3*
FA	C18:1		*281.1*		^{13}C-FA	C18:1		*299.2*
LPA	C14:0		*381.0*		^{13}C-LPA	C14:0		*395.0*
LPA	C14:1		*379.1*		^{13}C-LPA	C14:1		*393.2*
LPA	C16:0		*409.1*		^{13}C-LPA	C16:0		*425.1*
LPA	C16:1		*407.1*		^{13}C-LPA	C16:1		*423.1*
LPA	C18:0		*437.1*		^{13}C-LPA	C18:0		*455.1*
LPA	C18:1		*435.1*		^{13}C-LPA	C18:1		*453.1*
PA (DP)	16:0	16:0	*647.4*		^{13}C-PA (DP)	16:0	16:0	*679.5*
PA	16:0	16:1	*645.4*		^{13}C-PA	16:0	16:1	*677.5*
PA	16:0	18:0	*675.4*		^{13}C-PA	16:0	18:0	*709.5*
PA (PO)	16:0	18:1	*673.4*		^{13}C-PA (PO)	16:0	18:1	*707.5*
PA	16:1	16:1	*643.4*		^{13}C-PA	16:1	16:1	*675.5*
PA	16:1	18:0	*673.4*		^{13}C-PA	16:1	18:0	*707.5*
PA	16:1	18:1	*671.4*		^{13}C-PA	16:1	18:1	*705.5*
PA	18:0	18:0	*703.4*		^{13}C-PA	18:0	18:0	*739.5*
PA	18:0	18:1	*701.4*		^{13}C-PA	18:0	18:1	*737.5*
PA (DO)	18:1	18:1	*699.4*		^{13}C-PA (DO)	18:1	18:1	*735.5*
PG (PO)	16:0	18:1	*747.4*					

Supplementary Table 9. Genes used for cell-free protein synthesis.

#	Gene Name	Explanation
1	*plsX*	The gene for PlsX of *E. coli* K12 with silent mutations at the 5' end
2	*plsY*	The gene for PlsY of *E. coli* K12 with codon optimization for *E. coli* expression
3	*plsC*	The gene for Wildtype PlsC of *E. coli* K12
4	*plsX*-6His	The gene for PlsX(#1) with six histidines added at the C-terminus
5	*plsY*-6His	The gene for PlsY(#2) with six histidines added at the C-terminus
6	*plsC*-6His	The gene for PlsC(#3) with six histidines added at the C-terminus
7	*sfgfp-plsX*$_{wt}$	Fusion gene for sfGFP-PlsX(wildtype)
8	*plsY*$_{wt}$-sfgfp	Fusion gene for PlsY(wildtype)-sfGFP
9	*sfgfp*	The gene for sfGFP (super folder green fluorescent protein)

Supplementary Table 10. Template DNA and primers set for the In-Fusion cloning (or the 2-step PCR for Linear Template).

#	Gene Name	Vector /(Linear)	Primers for **Insert** PCR /(First PCR)	Primers for **Vector** PCR / (Second PCR)	Source of DNA
1	*plsX*(wildtype)	pET28a	• PURE_X_side_FW<br>• plsX_side_RV	• PURE_pET_RV<br>• Pet-X/Y-FW	Cell, pET28a
2	*sfgfp-plsX*(wildtype)	pET28a	• sfGFP_SG-FW<br>• sfGFP_SG-RV	• X_vector68-FW<br>• PURE_pET_RV	#11, #1
3	*plsX*	Linear	• Xmut_FW<br>• T7t	• Universal primer<br>• T7t	#1
4	*plsX*-6His	Linear	• T7p<br>• Xmut_His_RV	non	#3
5	*plsY*(wildtype)	pET28a	• plsY_side_FW<br>• plsY_side_RV	• PURE_pET_RV<br>• Pet-X/Y-FW	Cell, pET28a
6	*plsY*(wildtype)-sfgfp	pET28a	• SG_sfGFP-FW<br>• SG_sfGFP-RV	• Pet-X/Y-FW<br>• Y_vector35-RV	#11, #5
7	*plsY*	Linear	• Yopt-FW<br>• Yopt-RV	• Universal primer<br>• Yopt-RV	Synthetic DNA
8	*plsY*-6His	Linear	• T7p<br>• Yopt-His-RV	non	#7
9	*plsC*		non	non	Kuruma *et al.* [3]
10	*plsC*-6His	Linear	• T7p<br>• PlsC_His_RV	non	#9
11	*sfgfp*	pET32b	• non	non	Berhanu *et al.* [4]

Supplementary Text 1

Calculation to estimate the number of FA molecules synthesized inside GUV required for self-reproduction

When vesicle has 10 µm radius, surface area (S) of the vesicle is

(S) = 1256.64 µm^2 by the formula (**A**)

Considering a vesicle consists of the inner and outer leaflets, the total (S) value is

2(S) = 2513.27 µm^2

If the vesicle is formed with POPC, the number of POPC molecules (N_{popc}) forming the vesicle is

(N_{popc}) = 4E+09 by the formula (**B)**

Here, 0.66 nm^2 was used as the surface area (*a*) of POPC.

Because phospholipid (i.e., POPC) consists of two fatty acid chains, the number of N_{POPC} can be converted to the number of fatty acid molecules (N_{FA}) forming the vesicle as

(N_{FA}) = 8E+09

When radius of vesicle is 10 µm, internal volume (V) of the vesicle is

(V) = 4188.79 µm^3 by the formula (**C**)

This can be converted as

(V_{nL}) = 4.19E-03 nL

If the fatty acid synthesis system works within this volume, the number of *de novo* synthesized fatty acid molecule ($N_{in\ vesicle}$) is

($N_{in\ vesicle}$) = 8E+08 by the formula (**D**)

Here, 3E-04 M (300 µM) was used as concentration of FA (C_{FA}) synthesized in the vesicle.

If all of the internally synthesized *de novo* FA localized onto the mother vesicle membrane, this means 9.9 % of lipids forming the mother vesicle is synthesized from inside.

Formula A: $S=4\pi r^2$

Formula B: $N=2(S)/a$

Formula C: $V=4\pi r^3/3$

Formula D: C_{FA} V_{nL} 6.02E+08

Vesicle radius	N_{FA}	$N_{in\ vesicle}$*	$(N_{in\ vesicle})/(N_{FA})\cdot 100$ (%)
5	1.9E+09	9.5E+07	5.0
10	7.6E+09	7.6E+8	9.9
15	1.7E+10	2.6E+09	14.9
20	3.0E+10	6.1E+09	19.9
25	4.8E+10	1.2E+10	24.8
30	6.9E+10	2.0E+10	29.8

*Yield: 300 μM,

Supplementary References

1. Yu, X., Liu, T., Zhu, F. & Khosla, C. In vitro reconstitution and steady-state analysis of the fatty acid synthase from Escherichia coli. *Proceedings of the National Academy of Sciences of the United States of America*. **108**, 18643-18648 (2011).
2. Smith, A. C. & Cronan, J. E. Evidence against translational repression by the carboxyltransferase component of Escherichia coli acetyl coenzyme A carboxylase. *Journal of bacteriology*. **196**, 3768-3775 (2014).
3. Kuruma, Y., Stano, P., Ueda, T. & Luisi, P. L. A synthetic biology approach to the construction of membrane proteins in semi-synthetic minimal cells. *Biochim Biophys Acta*. **1788**, 567-574 (2009).
4. Berhanu, S., Ueda, T. & Kuruma, Y. Artificial photosynthetic cell producing energy for protein synthesis. *Nat Commun*. **10**, 1325 (2019).
